# Evaluation of earlier versus later dietary management in long-chain 3-hydroxyacyl-CoA dehydrogenase or mitochondrial trifunctional protein deficiency: a systematic review

**DOI:** 10.1186/s13023-019-1226-y

**Published:** 2019-11-15

**Authors:** Hannah Fraser, Julia Geppert, Rebecca Johnson, Samantha Johnson, Martin Connock, Aileen Clarke, Sian Taylor-Phillips, Chris Stinton

**Affiliations:** 1 Warwick Medical School, University of Warwick, Coventry, CV4 7AL UK; 2 Faculty of Health and Life Sciences, Coventry University, Coventry, CV1 5RW UK; 3 Warwick Library, University of Warwick, Coventry, CV4 7AL UK

**Keywords:** Systematic review, Long-chain 3-hydroxyacyl-CoA dehydrogenase, Mitochondrial trifunctional protein deficiency, Inborn errors of metabolism, Long-term outcomes, Newborn screening

## Abstract

**Background:**

Mitochondrial trifunctional protein (MTP) and long-chain 3-hydroxyacyl-CoA dehydrogenase (LCHAD) deficiencies are rare fatty acid β-oxidation disorders. Without dietary management the conditions are life-threatening. We conducted a systematic review to investigate whether pre-symptomatic dietary management following newborn screening provides better outcomes than treatment following symptomatic detection.

**Methods:**

We searched Web of Science, Medline, Pre-Medline, Embase and the Cochrane Library up to 23rd April 2018. Two reviewers independently screened titles, abstracts and full texts for eligibility and quality appraised the studies. Data extraction was performed by one reviewer and checked by another.

**Results:**

We included 13 articles out of 7483 unique records. The 13 articles reported on 11 patient groups, including 174 people with LCHAD deficiency, 18 people with MTP deficiency and 12 people with undifferentiated LCHAD/MTP deficiency. Study quality was moderate to weak in all studies. Included studies suggested fewer heart and liver problems in screen-detected patients, but inconsistent results for mortality. Follow up analyses compared long-term outcomes of (1) pre-symptomatically versus symptomatically treated patients, (2) screened versus unscreened patients, and (3) asymptomatic screen-detected, symptomatic screen-detected, and clinically diagnosed patients in each study. For follow up analyses 1 and 2, we found few statistically significant differences in the long-term outcomes. For follow up analysis 3 we found a significant difference for only one comparison, in the incidence of cardiomyopathy between the three groups.

**Conclusions:**

There is some evidence that dietary management following screen-detection might be associated with a lower incidence of some LCHAD and MTP deficiency-related complications. However, the evidence base is limited by small study sizes, quality issues and risk of confounding. An internationally collaborative research effort is needed to fully examine the risks and the benefits to pre-emptive dietary management with particular attention paid to disease severity and treatment group.

## Introduction

Long-chain 3-hydroxyacyl-CoA dehydrogenase (LCHAD) deficiency and mitochondrial trifunctional protein deficiency (MTPD) are rare autosomal recessive fatty acid β-oxidation disorders. Combined, they have an estimated prevalence of 1.02 per 100,000 live births worldwide [1]. MTP deficiency can be caused by either mutations in the HADHB gene or the HADHA gene, whilst LCHAD deficiency is only caused by mutations in the HADHA gene [2]. The HADHA gene encodes for the alpha subunit of the mitochondrial trifunctional protein (MTP) which is composed of four alpha and four beta subunits and which acts as a catalyst in three activities (as a hydratase, a dehydrogenase, and a thiolase) in the mitochondrial β-oxidation of long-chain fatty acids. In LCHAD deficiency (LCHADD), mutations occur within the alpha subunit of the LCHAD enzyme, with normal activity in the other two MTP enzymes. In MTP deficiency, mutations result in deficient activities in the two other MTP enzymes (long-chain enoyl-CoA hydratase and long-chain 3-oxoacyl-CoA thiolase) [3].

Common signs and symptoms of LCHAD/MTP deficiencies include fatigue, rhabdomyolysis and hypoketotic hypoglycaemia. Long-term complications include cardiomyopathy, organ failure and death. Clinical presentations of LCHADD/MTPD are variable, even in cases with the same underlying mutation [4]. Three clinical phenotypes have been described: (1) an early-onset severe form which presents from birth/a few days after birth and can result in sudden-infant death from cardiomyopathy or organ failure, (2) an infant-onset form which is often induced by infection and which causes, for example, hypoketotic hypoglycaemia, and (3) a later-onset myopathic form which is induced by exercise or illness and often presents as muscular problems, fatigue and rhabdomyolysis [5–7]. It has been suggested that people with MTP deficiency are more likely to have the early-onset severe form than those with LCHADD [8, 9]. Conversely, people with LCHADD are more likely to have the infant-onset form than those with MTP deficiency [10]. There may also be differences in long-term outcomes between the two conditions; retinopathy may be more common in people with LCHADD [11, 12] and peripheral neuropathy more common in people with MTPD [12].

Treatment for LCHADD and MTPD consists of a strict dietary management, which can include eating frequently, a low-fat and high-carbohydrate food plan, and/or taking supplements such as medium-chain triglycerides (MCT) [6]. It has been suggested that earlier treatment might result in better long-term outcomes than later treatment [6, 13]. Newborn screening is one method by which earlier diagnosis can be achieved. LCHAD/MTP deficiencies are already included in the newborn screening programmes of European countries such as Germany, Austria, Spain and Denmark [8, 13–15] as well as across North America [16, 17]. A key criterion to assess screening programmes is that there should be evidence that treatment at a *pre-symptomatic* (rather than just ‘early’) stage leads to better outcomes for individuals compared to those who are clinically detected following the onset of symptoms. To date, no systematic reviews have been undertaken which synthesise and quality appraise the evidence on detection and age at treatment initiation and their effects on long-term outcomes of LCHADD/MTPD patients. Therefore, the aim of this review is to investigate whether pre-symptomatic dietary management (following universal newborn screening, cascade testing due to previously affected sibling(s), or incidental detection) provides better long-term outcomes for patients with LCHAD/MTP deficiencies than later dietary management (after symptomatic presentation).

## Methods

The protocol is registered at the PROSPERO International Prospective Register of Systematic Reviews (registration number CRD42018094356).

### Search strategy

Systematic literature searches were undertaken in the following electronic databases: Web of Science (Core Collection), Medline (Ovid), Medline In-Process & Other Non-Indexed Citations (Ovid), Embase (Ovid), and the Cochrane Library (Wiley). We searched for terms relating to the condition such as MTP and LCHAD deficiencies as well as general terms such as fatty acid oxidation disorders and inborn errors of lipid metabolism (the full electronic search strategy can be found in Additional file 1). We also examined the reference lists of included studies and previous relevant systematic reviews. The search was conducted on 23rd April 2018. No date limits or language limits were applied.

### Eligibility criteria

We included articles that investigated people with genetically confirmed LCHADD or MTPD comparing any outcome after dietary management and other nutritional strategies (e.g. MCT supplementation) following (1) pre-symptomatic detection by screening (universal newborn screening, cascade testing or incidental detection) with (2) treatment following presentation with symptoms (either before or after the screening period).

We included any study design in humans that reported comparative data. Non-human studies, letters, editorials, communications, grey literature and conference abstracts were excluded. Studies of fatty acid β-oxidation disorders where data from people with mitochondrial trifunctional protein disorders could not be separated from data on other fatty acid oxidation disorders (e.g. multiple acyl-CoA dehydrogenase and very long-chain acyl-CoA dehydrogenase deficiencies) and studies where more than 10% of the sample did not meet our inclusion criteria, were also excluded. Systematic reviews were excluded but their references were checked for inclusion.

### Study selection and data extraction

The titles, abstracts, and full texts of papers were assessed independently by two reviewers. Data were extracted by one reviewer and checked by a second using a piloted electronic data collection form (Additional file 2). Disagreements were resolved through discussion, with the involvement of a third reviewer when required.

### Quality appraisal

Quality appraisal was undertaken independently by two reviewers; disagreements were resolved via consensus or a third reviewer. We used the Effective Public Health Practice Project (EPHPP) quality assessment tool for quantitative studies [18]. The EPHPP has six domains: selection bias, study design, confounders, blinding, data collection methods, withdrawals and dropouts. Each study is given an overall rating for quality of weak (two or more weak domains), moderate (one weak domain) or strong (no weak domains) [19].

### Data summary and synthesis

A narrative synthesis of study characteristics and outcomes is provided for all included studies.

There were differences in how ‘early’ was defined across studies. In the majority of cases ‘early’ was defined as screen-detected and asymptomatic, and ‘late’ was defined as clinically detected after presenting with symptoms. However, there is a subgroup of patients who present with symptoms at the time of screening. Studies varied in whether they included this group within the ‘screened’ group, or within a ‘symptomatic at diagnosis’ group. Three planned a priori follow up analyses were undertaken to address concerns about the applicability of including pre-screening symptomatic patients in the screen-detected group as well as possible confounding factors. These follow up analyses considered different subsets of the available individual patient data of the included articles:

(1) Asymptomatically vs symptomatically detected patients.

In this comparison, we allocated cases who were symptomatic within the first few days of life (so at the time of NBS screening) to the ‘symptomatically detected’ group and compared them to patients who were ‘asymptomatic’ at the time of NBS screening or cascade testing due to previously affected siblings. This may bias findings in favour of screening as these early symptomatic cases might have a more severe spectrum of disease.

(2) Screened vs unscreened patients.

In this comparison, the ‘screened’ group includes all patients identified via NBS screening (irrespective of being symptomatic at the time of screening or not) and patients identified via cascade testing due to previously affected siblings. Allocating the severe cases who have symptoms at the time of newborn screening to the ‘screened’ group biases against screening because in current practice these patients would undergo diagnostic testing anyway, so they would not actually benefit from universal newborn screening. In addition, the comparison might be biased in favour of screening due to most of the ‘unscreened’ patients being born prior to implementation of universal newborn screening and therefore being older than screened patients and experiencing a historical health care pathway.

(3) People who were asymptomatic at screening, symptomatic at screening, and those who were clinically detected in the absence of screening or who were clinically detected following false negative screening results.

To allow for the potential bias of including the severe forms of the diseases which present prior to screening, the third analysis analysed the three possible groups separately - asymptomatic at screening, symptomatic at screening and those with late clinical diagnosis due to symptoms.

In the follow up analyses, frequencies of complications between the groups were compared using the chi-square test; in cases of expected values smaller than 5, a Fisher’s exact test was used. All chi-square and Fisher’s exact tests were performed in IBM SPSS Statistics 24. Forest plots were prepared using Stata version 15.0 (Statacorp, College Station, TX, USA) with the metaprop command [20].

## Results

### Searching, sifting, and sorting

**Fig. 1:**
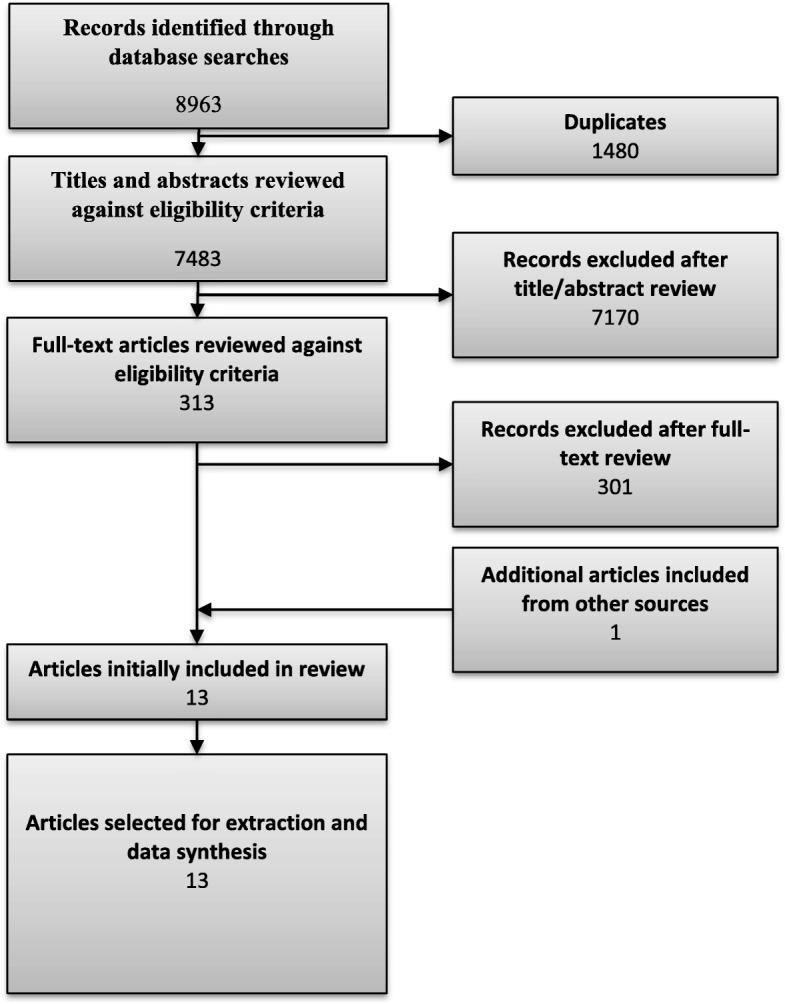
PRISMA flow diagram of records through the systematic review

Database searches yielded 7483 results, of which 313 full texts were assessed, and 12 were judged to be relevant to this review. An additional article was identified from a search for a related review all other references raised by this search were checked and none were deemed eligible for inclusion. Overall, 13 articles were ultimately included. Details regarding exclusions at each stage can be found in the PRISMA diagram (Fig. 1). Reasons for exclusions of full texts can be found in Additional file 3.

### Characteristics of included studies

The main characteristics of included studies are summarised in Table 1. Thirteen papers (reporting on 11 patient groups) compared outcomes for screen-detected and early-treated patients versus unscreened and later-treated LCHADD/MTPD patients [6, 9, 13, 15, 21–29]. The number of LCHADD/MTPD patients included per analysis ranged from five people from a single clinic in Utah, USA [26] to 59 from two centres in Poland [29]. A total of 174 people with LCHAD deficiency, 18 people with MTPD and 12 people with undifferentiated LCHAD/MTPD were included across all the studies.

**Table 1:** Characteristics of included studies

Study		Study design	Participants	Treatment
Swedish Cohort studies	Fahnehjelm 2008 [21]	Cohort study Average follow-up time: median 7.5 years (range 2.3–14.8 years) Study period: Not reported Study setting: Karolinska University Hospital and Uppsala University Hospital, Sweden Number of centres: 2	*n* = 10, 10/10 LCHADD. Asymptomatic screened: *n* = 1 (cascade testing). Age of diagnosis/treatment: First days of life Clinical presentation with symptoms: *n* = 9 Clinical symptoms (S) but no acute illness: *n* = 4 Severe symptoms (SS) (elevated liver enzymes and cardiomyopathy and /or seizures): *n* = 5 Age of diagnosis/treatment: 0-1 m (*n* = 1), 1-6 m (*n* = 2), > 6 m (*n* = 6)	All patients received a dietary treatment of low fat intake and essential fatty acid supplementation. All children also received DHA. Compliance of treatment is not reported.
	Fahnehjelm 2016 [22]	Cohort study. Prospective and retrospective data collection Average follow up time: median 15 years (range 3–26 years). Time period/study duration: Not reported Patients diagnosed between 1990 to using the same treatment guidelines Study setting: Karolinska University Hospital and Uppsala University Hospital, Sweden Number of centres: 2	*n* = 12, 12/12 LCHADD. Asymptomatic screened *n* = 3 (2 by NBS, 1 unspecified). Age of diagnosis/treatment: First days of life Clinical presentation with symptoms: *n* = 9 Clinical symptoms (S) but no acute illness: *n* = 4 Severe symptoms (SS) (elevated liver enzymes and cardiomyopathy and /or seizures): *n* = 5 Age of diagnosis/treatment: 0-1 m (*n* = 1), 1-6 m (*n* = 2), > 6 m (*n* = 6)	All patients received a dietary treatment of low fat intake and essential fatty acid supplementation. 11/12 had DHA. 8/12 continuous night feeds. Dietary compliance: Asymptomatic screened: all acceptable S clinical: 1/4 (25%) poor, 3/4 (75%) acceptable SS clinical: 3/5 (60%) poor, 2/5 (40%) acceptable
	Haglind 2013 [23]	Cohort study, retrospective data collection of medical reviews Time period/study duration: Not reported Patients aged up to 20 years Study setting: Karolinska University Hospital and Uppsala University Hospital, Sweden Number of centres: 2	*n* = 10, 10/10 LCHADD. Asymptomatic screened: *n* = 1 (cascade testing). Age of diagnosis/treatment: 2 days Clinical presentation with symptoms: *n* = 9 Clinical symptoms (S) but no acute illness: *n* = 4 Severe symptoms (SS) (elevated liver enzymes and cardiomyopathy and / or seizures): *n* = 5 Age of diagnosis/treatment: mean 6.1 months (up to 13 m)	8/10 received DHA. 9/10 had a PEG with continuous night feeds. 9/10 received MCT fat, vitamins, minerals, and trace elements. Fasting limited to 3–4 h. 2/10 uncooked corn starch. 1 had carnitine deficiency so given carnitine supplements of 25-50 mg/kg/day. Did not record compliance.
Immonen 2016 [24]		Prospective cohort (followed prospectively but using diagnosis data from retrospectively collected hospital records). Comparison with historical cohort (24/28 diagnosed post mortem) Follow-up time (age of patients at the end of the study): 1–11 years Time period: 1997–2010 Study setting: Hospitals in Finland Number of centres: NR	*n* = 16, 16/16 LCHADD. Asymptomatic screened: *n* = 1 (cascade testing) Age at treatment: Birth Symptomatic clinical: *n* = 15 Age at presentation: Birth to 0.42 years (~ 5 months). Mean 0.27 years. Age at diagnosis: Up to 6 months	Age at treatment initiation: 1–30 days of diagnosis All patients in both groups received a low-fat diet, MCT, essential fatty acids and DHA (this was 10 clinical patients as the remainder were not alive). Fasting of more than 3 or 4 h avoided in infancy and childhood. Good compliance of diet in 9/11 patients.
Sperk 2010 [9]		Case series (6 cases) – clinical histories obtained from referring physicians Maximum follow up until age 5 years Study duration: 3 years Study setting: University Children’s hospital, Düsseldorf, Germany Number of centres: 1	*n* = 6, 3/6 LCHADD, 3/6 MTPD. Asymptomatic screened: *n* = 3 (1 LCHADD, 2 MPTD) All diagnosed and began treatment 4–5 days of age Symptomatic screened: *n* = 3 (2 LCHADD, 1 MTPD)	Type of treatment not reported. Dietary adherence not reported.
Karall 2015 [13]		Retrospective cohort (review of medical records) Study duration: Birth – October 2013 Follow-up time: Range 0.9–15.4 years (median 7.8 years, mean 6.9 years) Study setting: Metabolic Centres in Austria (Graz, Innsbruck, Salzburg, Vienna) and Germany (Munich) Number of centres: 5	*n* = 14, 14/14 LCHADD. Asymptomatic screened: *n* = 6 Age at diagnosis median (range): 1.5 days (1–10 days) Symptomatic screened: *n* = 3 Age at diagnosis median (range): 15 days (15 days) Pre-NBS clinical: *n* = 3 Age at diagnosis median (range): 5 months (3–20 months) False negative (FN) screen clinical: *n* = 2 Age at diagnosis median (range): 4.5 months (4–5 months)	All cases received low-fat diet and MCT supplements. Triheptanoin was used in 2/3 symptomatic screened, 1/3 pre- NBS and 1/2 FN NBS. Essential fatty acids (walnut oil) were given to 2/3 pre-NBS clinical cases. DHA given to 6/6 asymptomatic screened, 1/3 symptomatic screened, 2/3 pre-NBS and 1/2 in FN NBS. PEG used in 1/6 asymptomatic screened, 1/3 pre-NBS and 1/2 FN NBS Dietary compliance not reported.
Spiekerkoetter 2009 [6]		Retrospective cohort (questionnaire study) Follow-up time: NR Study setting: Metabolic Centres, Germany/Switzerland/ Austria/the Netherlands Number of centres: 18	*n* = 75, Relevant to this review: *n* = 27, 20/27 LCHADD, 7/27 MTPD. Screened: *n* = 10 (7 LCHADD, 3 MTPD). Age at diagnosis: Newborn, 7/10 symptomatic at NBS. Clinically diagnosed: *n* = 17 (13 LCHADD, 4 MTPD). Age at diagnosis: LCHADD: Median 5 months (range 3 days – 11 years) MTPD: median 1 year (range 1 day - 4.5 years)	Data available on LCT and MCT in 14/27 and 17/27 of patients: 13/14 LCT intake restricted, 17/17 supplemented with MCT, 11/14 received additional carbohydrates, 2/14 on continuous overnight nasogastric tube feeding, 1 supplemented with DHA, 1 receiving Triheptanoin. Dietary compliance not reported.
Boese 2016 [25]		Retrospective case series (cohort) Time period: 20/9/1994–18/8/2015 Follow up period: Median 5.6 years (range 0.3–20.2 years) Study setting: Oregon Health and Science University (OHSU) Casey Eye Institute, USA Number of centres: 1	*n* = 21, 18/21 LCHADD, 3/21 MTPD. Screened: *n* = 7 (6 LCHADD, 1 MTPD). Age at diagnosis: newborn, 1 LCHADD case symptomatic at screening. Clinical: *n* = 14 (12 LCHADD, 2 MTPD). Age at diagnosis: median 4.5 months (range 1 day – 3 years)	A diet low in long-chain fatty acids and supplementation with MCT. All subjects and/or guardians were counselled to avoid fasting. Some subjects were prescribed oral carnitine supplements. Dietary intake assessed by 24 h recall.
De Biase 2017 [26]		Retrospective cohort (chart review) Average follow-up time: nearly 10 years (mean 9.2 years, SD 5.9 years) Study setting: Metabolic Clinic University of Utah, USA Number of centres: 1	*n* = 5, 4/5 LCHADD, 1/5 MTPD. Asymptomatic screened: *n* = 1 (1 LCHADD) Age at diagnosis: birth (NBS) Symptomatic screened: *n* = 2 (1 LCHADD, 1 MTPD). Age at diagnosis: birth (NBS) Symptomatic clinical: *n* = 2 (2 LCHADD) Age at diagnosis: Median 5 months (range 4–6 months)	All patients received low-fat diet, MCT, essential fatty acids and carnitine. All patients bar symptomatic clinical treated received cornstarch. Both screened symptomatic cases and 1/2 clinical symptomatic patients received DHA. One late treated patient is noted to have followed dietary therapy with variable compliance.
Gillingham 2017 [27]		Double blind parallel RCT (retrospective data collected on time of diagnosis) Follow-up time NR Study setting: Oregon Health and Science University and University of Pittsburgh, USA Number of centres: 2	*n* = 24 Included for this review: *n* = 12. 8/12 LCHADD, 4/12 MTPD. Asymptomatic screened: *n* = 7 (5 LCHADD, 2 MTPD). Age at diagnosis/treatment (range): newborn (0-2 m). Symptomatic clinical: *n* = 5 (3 LCHADD, 2 MTPD). Age at diagnosis/treatment (range): Infancy (2 m-2y) *n* = 4 or childhood (2y-10y) *n* = 1.	All patients in the asymptomatic and symptomatic groups received a low-fat diet and MCT. 3/7 from the asymptomatic group received Triheptanoin (2 LCHADD, 1 MTP). 4/7 from the asymptomatic group received Trioctanoin (3 LCHADD, 1 MTP). 4/5 from the symptomatic group received Triheptanoin (2 LCHADD, 2 MTP) 1/5 from the symptomatic group received Trioctanoin (1 LCHADD) Dietary compliance not reported.
Kang 2018 [28]		Retrospective cohort Follow-up time: ~ 10 years Time period: May 2002 – February 2016 Study setting: Department of Medical Genetics, Asan Medical Center Children’s Hospital, Seoul, Korea Number of centres: NR	*n* = 22 Included for this review: *n* = 7 LCHADD/MTPD not differentiated but genotypes are suggestive of MTPD. Asymptomatic screened: *n* = 1 Symptomatic clinical: *n* = 6	Screened patient educated to avoid prolonged fasting, MCT diet with long-chain fat restriction Dietary compliance not reported.
Lund 2012 [15]		Case-control study Follow-up time: Range 2–109 months Time period: Feb 1st 2002 – Mar 31st 2011 (trial 2002–2009) Study setting: Statens Serum Institut, Copenhagen. Cases from Denmark, Faroe Islands and Greenland Number of centres: 1	*n* = 5 LCHADD and MTPD not differentiated. Asymptomatic screened: *n* = 3 Age at diagnosis/treatment median (range): 6 days (1d-5d) Symptomatic clinical: *n* = 2 Age at diagnosis/treatment median (range): 4.5 months (4.5 months)	Type of dietary management not reported Dietary compliance not reported.
Sykut-Cegielska 2011 [29]		Retrospective cohort Follow-up time: Up to 17 years Time period: 1992–2009 Study setting: Children’s Memorial Health Institute and Institute of Mother and Child Warsaw, Poland Number of centres: 2	*n* = 59, 58/58 LCHADD, 1 not analysed. Asymptomatic screened: *n* = 15 Age at diagnosis/treatment: Median 14 days (range 4 days - 8 weeks) Detected by cascade testing, TMS pilot screening or by chance from PKU screening. Symptomatic clinical: *n* = 44 Age at diagnosis/treatment: median (range): 6 m (1 m-18y1m) Group includes all those tested due to suspicions of metabolic disorders post mortem and diagnoses established abroad.	Type of dietary management not reported. Dietary compliance not reported.

*FN* False Negative, *LCHADD* Long-chain 3-hydroxyacyl-CoA dehydrogenase deficiency, *MCT* Medium-chain triglyceride, *MTPD* Mitochondrial Trifunctional Protein Deficiency, *PKU* Phenylketonuria, *NBS* Newborn Blood Spot, *PEG* Percutaneous endoscopic gastrostomy, *S* symptomatic, *SS* severe symptomatic

Seven of the eleven included studies were retrospective cohort studies [6, 9, 13, 25, 26, 28, 29]. Three studies (reported in five papers) were prospective studies [15, 21–24] and one was a randomised controlled trial (RCT) for a drug treatment which has been analysed as a cohort study in this review [27]. The shortest study duration was three years [9] and the longest period of follow up was up to 17 years [29]. One study did not report follow up time [6]. Type of dietary management was not specified in three of the studies [9, 15, 29]. In the remaining eight studies all received a diet of low fat intake with essential fatty acid supplementation. Whether patients were given docosahexaenoic acid (DHA), carnitine, or MCT (e.g. Triheptanoin) supplements varied across studies and across patients within studies. Five papers (reporting on three patient groups) reported on dietary compliance [21–24, 26].

### Quality appraisal

**Fig. 2:**
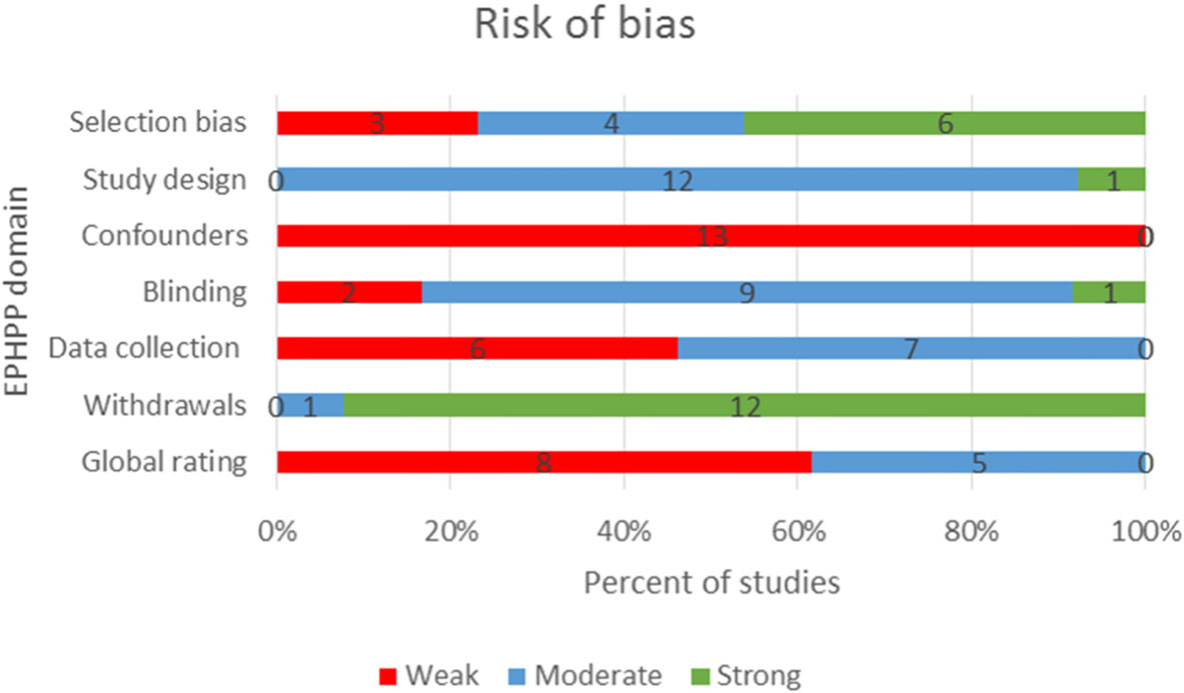
Risk of bias – authors’ judgements using the EPHPP tool

The quality assessment of included studies can be found in Fig. 2 and Additional file 4. Overall, the methodological quality was judged as weak in seven studies (8 papers), with two or more domains receiving a weak rating [6, 9, 21, 22, 24, 26, 27, 29]. The five remaining studies were rated as moderate, with one domain receiving a weak rating [13, 15, 23, 25, 28].

There was a high risk of selection bias in three studies [6, 26, 27]. In all three studies it was unclear if the individuals selected to participate in the study were representative of the target population as it was not specified if all clinic patients or a random sample were included. Study design quality was rated as moderate in all studies for having cohort designs including one RCT of a treatment which was effectively a cohort study for our research question.

All studies were of weak methodological quality in relation to confounding, since important factors (i.e. the presenting form of LCHAD/MTP deficiency, genotype, compliance with treatment, co-treatment) were not controlled for in study design or analysis. The quality of the blinding methods was rated as moderate across all studies [6, 9, 13, 15, 21–29]. One of these studies was an RCT that used double blinding, but for randomisation to a treatment drug not for method of detection [27]. In all other studies the outcome assessor knew whether the participants had been screened or clinically detected, but the participants were not aware of the research questions. Data collection methods were of weak methodological quality in six of the eleven studies with the validity and reliability of the tools used not specified [6, 9, 24, 26, 27, 29]. There was strong methodological quality in the ‘withdrawals and drop-outs’ domain in ten out of the eleven studies. The only study to be rated as moderate quality within this domain reported full data on only 10/37 included LCHADD/MTPD patients [6].

### Outcomes

This section compares outcomes following ‘early’ vs ‘late’ treatment initiation as defined in the original articles. Therefore, the allocation of the cases with symptoms at the time of NBS screening can differ between the studies. A wide range of outcomes was reported across the studies. We report a narrative synthesis of the three main groups of outcomes which were reported across the studies (mortality, cardiac problems and liver problems). Details on other outcomes are available in Table 2 and Additional file 5.

**Table 2:** Outcomes of included studies

Study		Mortality	Cardiac problems	Liver problems	Other
Immonen 2016 [24]		Asymptomatic screened: 0/1 Symptomatic clinical: 6/15 (40%) Age at death for 5/6 mean 8.5 m. Median 5 m (range 3 m-2y)	Cardiomyopathy at diagnosis Asymptomatic screened: 0/1 S clinical 6/10 (60%) Cardiomyopathy at the end of study period: Asymptomatic screened: 0/1 S clinical 4/10 (40%)	NR	Retinopathy Asymptomatic screened: 0/1 S Clinical: Mild: 7/10 (70%) Moderate: 1/10 (10%) Yes: 1/10 (10%) No: 1/10 (10%) Neuropathy Asymptomatic screened: 0/1 (not detected) S clinical: Mild: 2/9 (22.2%), Moderate: 1/9 (11.1%), None: 2/9 (22.2%), Not detected: 4/9 (44.4%)
Sperk 2010 [9]		Asymptomatic screened: 1/3 (33.3%) LCHADD patient, age at death: 3 months Symptomatic screened: 0/3	Cardiomyopathy Asymptomatic screened: 0/3 Symptomatic screened: 2/3 (66.7%) both cases MTPD	NR	Motor/muscular problems Asymptomatic screened: 0/3 Symptomatic screened: 1/3 (33.3%) MTPD case Hypoglycaemia Asymptomatic screened: 1/3 (33.3%) LCHADD case Symptomatic screened: 3/3 (100%)
Karall 2015 [13]		NR	Cardiomyopathy Asymptomatic screened: 1/6 (16.7%), median age 4 months Symptomatic screened: 1/3 (33%), median age 9 months Pre-NBS clinical: 3/3 (100%), median age 23 months (range 3–156 months) FN NBS clinical: 2/2 (100%), median age 4.5 months (range 4–5 months)	Hepatopathy Asymptomatic screened: 1/6 (16.7%), neonatally Symptomatic screened: 0/3 Pre-NBS clinical: 2/3 (66.7%), median age 13 months (range 3–23 months) FN NBS clinical: 2/2 (100%), median age 4.5 months (range 4–5 months)	Retinopathy Asymptomatic screened: 2/6 (33.3%), median age of onset 53 months (range 50–56 months) Symptomatic screened: 1/3 (33.3%), median age of onset 39 months Pre-NBS clinical: 3/3 (100%), median age of onset 24 months (range 23–108 months) FN NBS clinical: 2/2 (100%), median age of onset 40 months (range 38–42 months) Motor/muscular problems Psychomotor developmental normal in all patients
Spiekerkoetter 2009 [6]		Screened: 2/10 (20%), both MTPD, age at death median 5.5 days (range 3–8 days) Clinical: 6/17 (35.3%), 3 LCHADD, 3 MTPD; age at death median ~ 2 months (range 2 days – 4 years)	Cardiomyopathy Screened: 4/10 (40%) 1 LCHADD, 3 MTPD Clinical: 8/17 (47%) 7 LCHADD, 1 MTPD Arrhythmias Not reported in screened group. Clinical: 1/17 (5.9%)	Reye syndrome Screened: 3/10 (30%) 1 LCHADD, 2 MTPD Clinical: 6/17 (35.3%) All LCHADD	Retinopathy Screened: NR Clinical: 6/17 (35.3%) 5 LCHADD, 1 MTPD Neuropathy Screened: NR Clinical: 3/17 (17.7%) 2 LCHADD, 1 MTPD Hypotonia/Myopathy Screened: 4/10 (40%) 2 LCHADD, 2 MTPD Clinical: 14/17 (82.4%) 12 LCHADD, 2 MTPD Hypoglycaemia Screened: 4/10 (40%) 3 LCHADD, 1 MTPD Clinical: 15/17 (83.2%) 13 LCHADD, 2 MTPD
Boese 2016 [25]		NR	NR	NR	Best corrected visual acuity visit 1 Screened: 4/7 (57.1%) Central Steady and Maintained (CSM) (1 symptomatic case was CMS; 3 LCHADD) Clinical 4/14 (28.6%) CSM Best corrected visual acuity visit 2 Screened: 0/7 CSM Clinical: 2/17 CSM (11.8%) (1 LCHADD, 1 MTPD) Vision visit 1 (calculated by reviewers) Screened: 7/7 (100%) normal Clinical: 14/14 (100%) normal Vision visit 2 (calculated by reviewers)^e^ Screened: 7/7 (100%) normal Clinical: 2/14 impaired (14.3%) > 1 episode of rhabdomyolysis Screened: 6/7 (85.7%) Clinical: 13/13 (100%) Hypoglycaemia All LCHADD clinical cases presented with hypoketotic hypoglycaemia
De Biase 2017 [26]		NR	Arrhythmias Asymptomatic screened: 0/1 Symptomatic screened: 0/2 Symptomatic clinical: 1/2 (50%), 1 LCHADD	NR	Retinopathy Asymptomatic screened: 1/1 (100%), 1 LCHADD Symptomatic screened: 1/2 (50%), 1 LCHADD Symptomatic clinical: 2/2 (100%), 2 LCHADD Neural problems Asymptomatic screened: 0/1 Symptomatic screened: 0/2 Symptomatic clinical: 1/2 (50%), 1 LCHADD Myoglobinuria Asymptomatic screened: 0/1 Symptomatic screened: 0/2 Symptomatic clinical: 1/2 (50%), 1 LCHADD Hypoglycaemia at diagnosis Asymptomatic screened: 0/1 Symptomatic screened 2/2 (100%), 1 LCHADD, 1 MTPD Symptomatic clinical: 1/2 (50%), 1 LCHADD
Gillingham 2017 [27]		NR	Cardiac complications Asymptomatic screened: 0/7 Symptomatic clinical: 3/5 (60%), all 3 LCHADD	NR	NR
Kang 2018 [28]		Asymptomatic screened: 1/1 (100%) Age at death: 49 days Symptomatic clinical: 2/6 (33.3%) Median age at death (range): 6.5 days (4–9 days)	NR	NR	NR
Lund 2012 [15]		Asymptomatic: 0/3 Symptomatic clinical: 1/2 (50%) Age at death: 4 months	Cardiomyopathy Asymptomatic screened: 0/3 Symptomatic clinical: 2/2 (100%)	Hepatopathy Asymptomatic screened: 0/3 Symptomatic clinical: 1/2 (50%)	NR
Sykut-Cegielska 2011 [29]		Asymptomatic screened: 1/15 (6.7%) Age at death: 7 days Symptomatic clinical: 19/44 (43%) Age at death: Mean 23.95 m; median 6 m (range 4d-10y1m)	NR	NR	NR
Swedish Cohort studies	Fahnehjelm 2008 [21]	NR	NR	NR	Visual problems ERG findings Asymptomatic screened: Abnormal: 0/1 Pathological: 0/1 S clinical Abnormal: 2/4 (50%) Pathological: 2/4 (50%) SS clinical: Abnormal: 1/5 (20%)^a^ Pathological: 4/5 (80%) Photophobia Asymptomatic screened: 1/1 (100%) S clinical: 3/4 (75%) SS clinical: 4/4 (100%)^b^ Nyctalopia Asymptomatic screened: 0/1 S clinical: 0/4 SS clinical: 2/4 (50%)^c^ Psychomotor development Asymptomatic screened: Developmental delay (DD): 0/1 Severe DD: 0/1 S clinical: DD: 1/4 (25%) Severe DD: 0/4 SS clinical: DD: 3/5 (60%) Severe DD: 1/5 (20%) Neonatal hypoglycaemia Asymptomatic screened: 0/1 S clinical: 3/4 (75%) SS clinical: 4/5: (80%)
	Fahnehjelm 2016 [22]	NR	NR	NR	Visual problems ERG findings Asymptomatic screened: Subnormal: 1/2^d^ (50%) Pathological: 0/2 S clinical: Subnormal: 2/4 (50%) Pathological: 1/4 (25%) SS clinical: Subnormal: 1/5 (20%) Pathological: 4/5 (80%) Best corrected visual acuity Asymptomatic screened: no/mild visual impairment: 2/2 (100%) Missing data 1/3 (33.3%) S clinical: Moderate impairment: 1/4 (25%) No/mild impairment: 3/4 (75%) SS clinical: Blindness: 1/4 (25%) No/mild impairment: 2/4 (50%) Missing data: 1/5 (20%) Ocular fundi Asymptomatic screened: Normal: 1/3 (33.3%) Subnormal: 2/3 (66.7%) S clinical: Pathological: 4/4 (100%) SS clinical: Pathological 4/5 (80%) Severely pathological 1/5 (20%) Neural problems Epilepsia Asymptomatic screened: 0/3 S clinical: 1/4 (25%) SS clinical: 2/5 (40%) Hypoglycaemia Asymptomatic screened: 1/3 (33.3%) S clinical: 3/4 (75%) SS clinical: 4/5 (80%)
	Haglind 2013 [23]	NR	NR	NR	Hypoglycaemia Asymptomatic screened: 0/1 S clinical: 3/4 (75%) SS clinical: 4/5: (80%)

DD Developmental delay; ERG electroretinography test S symptomatic; SS severe symptomatic

^a^Progressive subnormal

^b^No information on one person

^c^Further visual measures are provided in the paper

^d^1 no ERG

eThe World Health Organization established criteria for low vision using the LogMAR scale. Low vision is defined as a best-corrected visual acuity worse than 0.5 LogMAR but equal or better than 1.3 LogMAR in the better eye. Blindness is defined as a best-corrected visual acuity worse than 1.3 LogMAR in the better eye. Normal defined as above 0.5

### Mortality

Mortality was reported as an outcome in six studies [6, 9, 15, 24, 28, 29]. Overall, mortality rates across all groups was 3/30 (10%) in the early treated groups compared to 30/83 (36%) in the late treated groups. In four of these studies, mortality rates were lower in the early-treated group (range 0–20%, 1/26 total) than in the later-treated groups (range 37.3–50%, 28/74 total) [6, 15, 24, 29]. In the remaining two studies mortality was lower in the later-treated group (range 0–33.3%, 2/9 total) than the early-treated group (range 33.3–100%, 2/4 total) [9, 28]. The median age at death across the studies was 28 days in the early-treated group (range 3 days–3 months) and 4 months (range 2 days–10 years 1 month) in the late-treated group.

### Heart related problems

Seven articles reported on heart related problems such as cardiomyopathy, arrhythmias or cardiac complications [6, 9, 13, 15, 24, 26, 27]. In all seven studies there were fewer heart problems in the early treatment group (range 0–40%, 5/31 of total patients) than the late treated group (range 25–100%, 20/32 of total patients). Median age at study end was reported in four of the studies [13, 24, 26, 27]. In the early groups the median age at study end ranged from 2 to 9 years, and in the later treatment group the median age at study end ranging from 2 to 20.5 years [13, 24, 26, 27]). However, the only study to report age at diagnosis of cardiomyopathy found the median age to be lower in pre-symptomatically detected patients (4 months) than in symptomatically presenting patients (4.5 months in the patients not detected by newborn screening, 9 months in those with symptoms at newborn screening, and 23 months in those diagnosed before the introduction of screening) [13].

### Liver related problems and Reye syndrome

Two studies reported on the incidence of liver problems [13, 15]. In both studies there were fewer instances of liver related problems in the early treatment group. In the first study there were 1/6 (16.7%) cases from the screen-detected group (median age 5.1 years at study end) with liver problems, whilst there were 4/8 (50%) cases in the later treatment group (median age 9.4 years at study end) [13]. In the second study, 0/3 people detected pre-symptomatically had liver related problems but both symptomatically presenting patients did (2/2, age of patients at the time of the study had liver problems (age at study end was not reported in this study)) [6]. Incidence of Reye syndrome was reported in one study [6]. There were slightly fewer cases of Reye syndrome in the early treatment group than the later treatment group [3/10 (30%) vs 6/17 (35.3%) respectively].

### Visual problems

Problems related to vision were reported in 5 studies across 7 papers [13, 21–26]. The studies reported on outcomes such as electroretinography (ERG) findings, best corrected visual acuity, ocular fundi findings and retinopathy. One study (across 2 papers) reported on ERG findings [21, 22]. One out of 2 (50%) individuals treated early had subnormal results and neither person had pathological results. Three out of 9 patients (33.3%) from the later treatment groups had subnormal results and 5 out of 9 patients (55.6%) had pathological findings. In the same study all early treatment patients had mild or no visual impairment (2/2), while in the later treatment groups one of 9 patients (11.1%) had moderate impairment and one out of 9 patients (11.1%) was blind. All people treated asymptomatically had either normal or subnormal (3/3) ocular fundi findings, and each patient in the later treatment group had either pathological or severely pathological findings (9/9) [22]. Three studies reported on retinopathy [13, 24, 26]. Two of the 3 studies found less retinopathy in the early treatment group (0–33.3%, 0/1 and 2/6) compared to 75–90% (6/8 and 9/10) showing mild to full retinopathy in the late treatment group. One study found 100% retinopathy in the early treatment group, though this group only included one person. This compares to 75% retinopathy in the later treatment group (3 out of 4 patients).

### Neurological problems

Neurological problems were reported in 4 studies [6, 22, 24, 26]. The reported outcomes were epilepsy, neuropathy and neurological symptoms. There were no instances of any neurological problems in any of the people who were treated early (*n* = 15 across the studies) compared to problems in every late treatment group [10/36 (27.7%) ranging from 17.7 to 33.3%].

### Motor and muscular problems

Muscular and motor problems were reported in six studies [6, 9, 13, 21, 25, 26]. The studies reported on psychomotor development, myopathy, episodes of rhabdomyolysis, and myoglobinuria. There were fewer motor and muscular problems in all early treatment groups compared to the late treatment groups across all studies (0–40%, 5 out of a total of 17 in the early group, compared to 25–82.4%, 21 out of a total of 38 in the late group).

### Pre-specified follow up comparisons

**Fig. 3:**
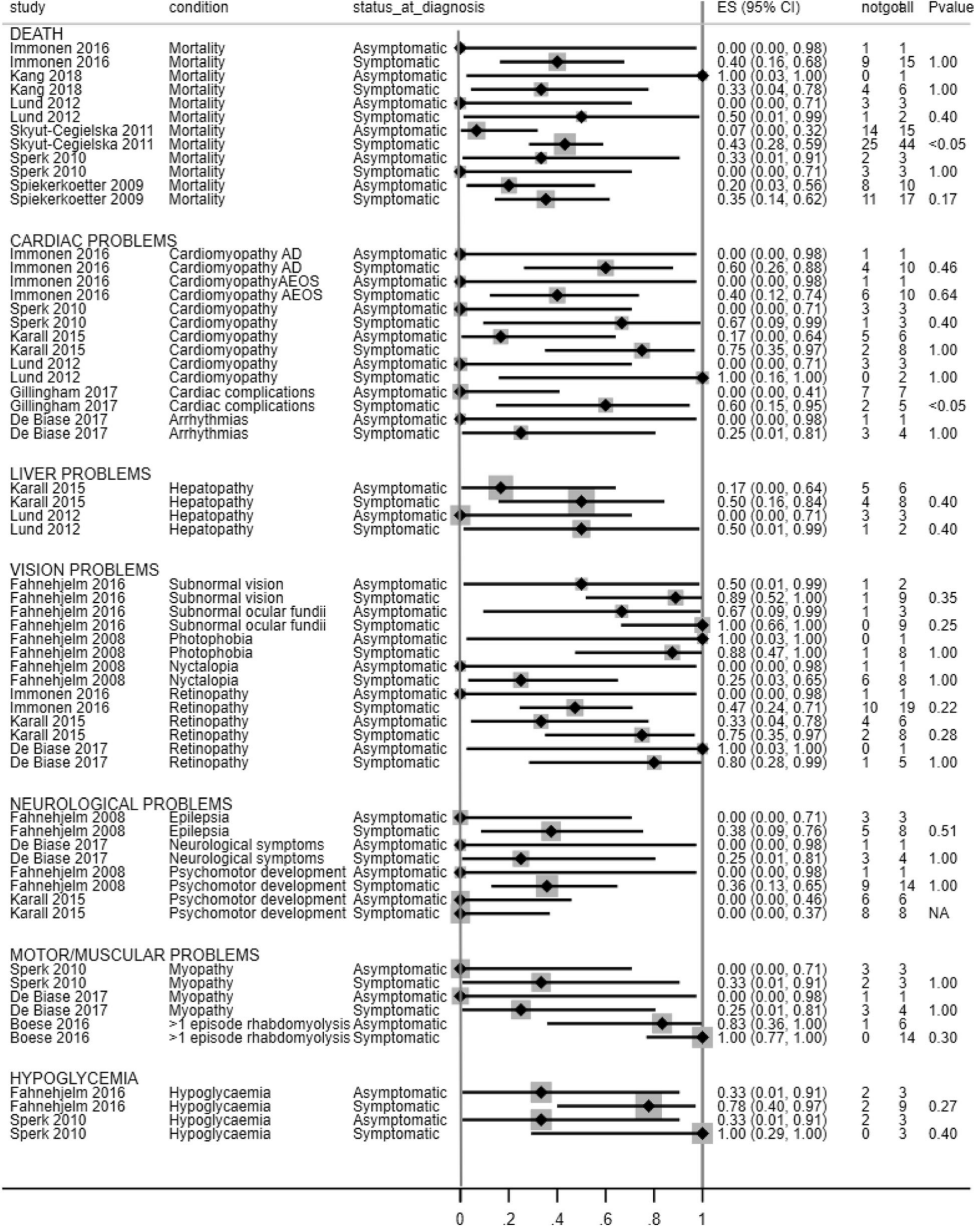
Forest plot showing mortality and incidence of cardiac and liver problems across symptomatically and asymptomatically detected patients per study (follow up analysis 1)

**Fig. 4:**
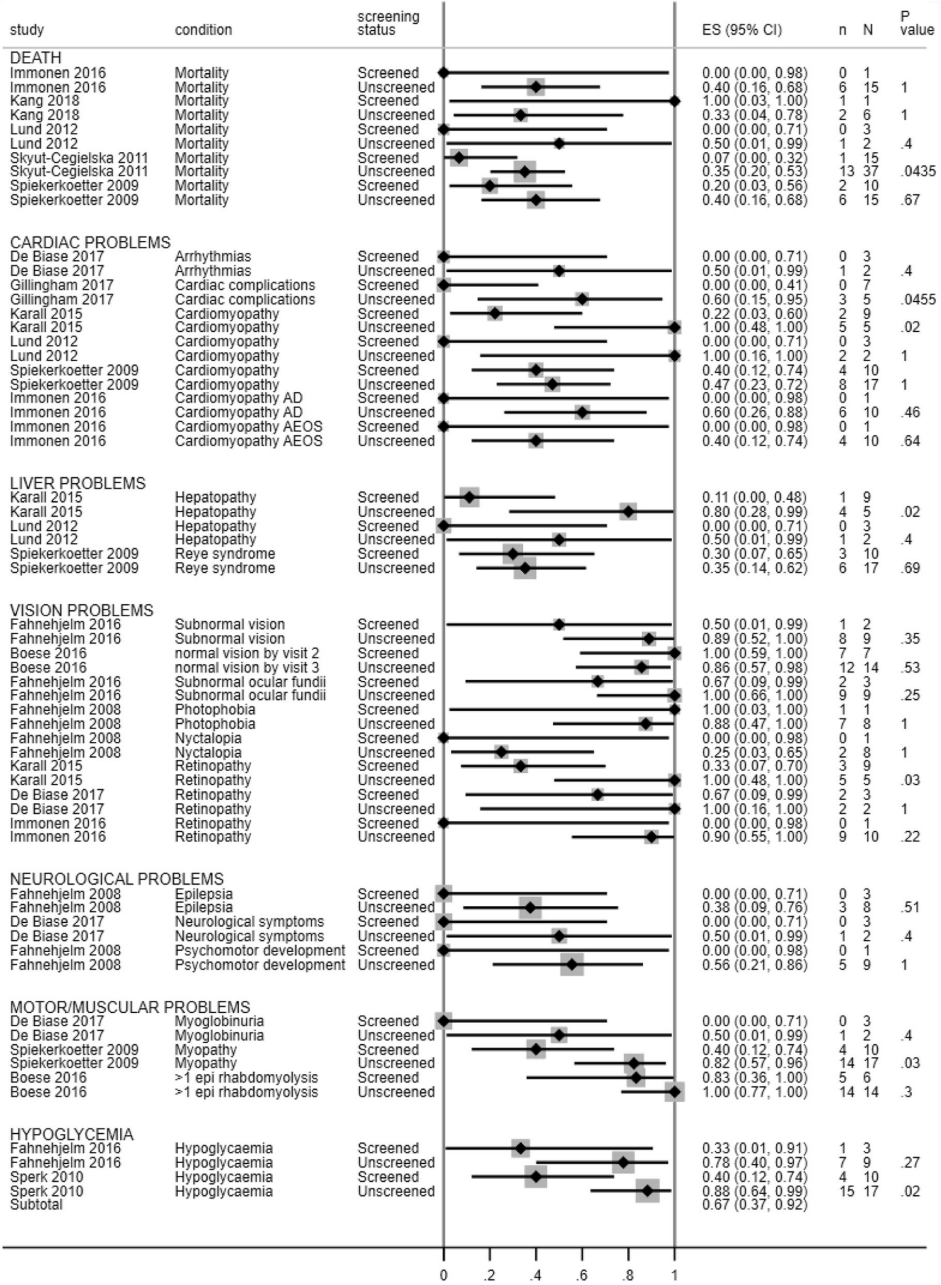
Forest plot showing mortality and incidence of cardiac and liver problems across screened and unscreened patients per study (follow up analysis 2)

**Fig. 5:**
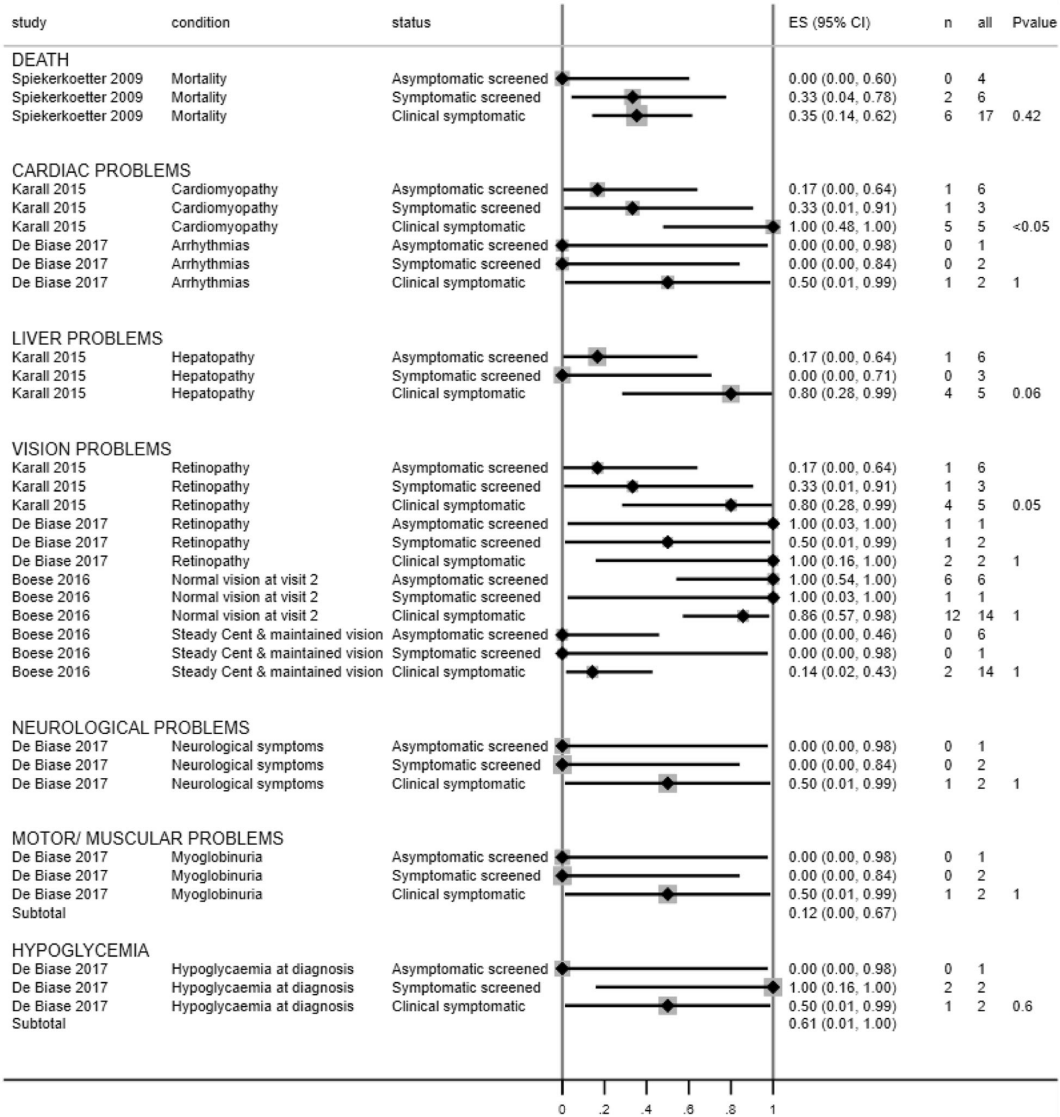
Forest plot showing mortality and incidence of cardiac and liver problems across symptomatic screened, asymptomatic screened and symptomatic clinically diagnosed patients per study (follow up analysis 3)

The following three follow up analyses considered different subsets of the available individual patient data. Results of the follow up analyses are presented in Figs. 3, 4 and 5 and in Additional file 5.

### Asymptomatically vs symptomatically detected patients

Individual patient data reported in eight papers were used for the follow up comparisons of outcomes in asymptomatically vs symptomatically detected patients [15, 21–24, 27–29]. Details are provided in Fig. 3 and Additional file 5. One patient group included cases diagnosed post mortem and cases with unknown method of diagnosis within the group with symptoms at diagnosis. For the purpose of these follow up analyses these cases were excluded (*n* = 7) [29].

There were no significant differences in the incidence of *liver, visual, neurological, motor or muscular problemss* between pre-symptomatically and symptomatically detected patients in the studies reporting data for these outcomes [9, 13, 15, 21–26].

There was no significant difference in *mortality rates* in 5/6 analysed patient groups reporting on asymptomatic versus symptomatically detected patients [6, 9, 15, 24, 28] . In the remaining study, significantly more deaths occurred in the symptomatically presenting patients (19/44, median age at death 1.82 years) than in the pre-symptomatically detected patients (1/15, *p* = 0.01, age at death 7 days) [29].

There was no statistically significant difference in the frequency of heart problems in 5/6 analysed patient groups [9, 13, 15, 24, 26]. In one study, significantly more cardiac complications occurred in the symptomatic group (3/5, median age at study end 9 years) than in the asymptomatic group (0/7, *p* < 0.05, median age at study end 17 years) [27].

### Screened vs unscreened patients

Follow up analyses were undertaken to see how grouping people by whether they were detected through screening (including cascade testing) or outside of screening affected outcomes. Details are provided in Fig. 4 and Additional file 5.

Among the five studies that reported on mortality [6, 15, 24, 28, 29], no statistically significant differences were found between the screened and unscreened groups in four studies [6, 15, 24, 28]. In the final study, there were significantly fewer deaths in the screened group (1/15, median age at death 7 days) than in the clinically detected unscreened group (13/37, median age at death 1.82 years, *p* < 0.05) [29].

Among the six studies reporting on heart problems [6, 13, 15, 24, 26, 27], no statistically significant differences were found between screened and unscreened groups in four of the studies [6, 15, 24, 26]. In the remaining two studies, one study found significantly fewer cases of cardiomyopathy in the screened group (2/9, median age 5.06 years) compared to the clinically detected unscreened group (5/5, median age 9.4 years, *p* = 0.02) [13]. The second study found significantly fewer cardiac complications in the screened group (0/7) compared to the unscreened group (3/5, p < 0.05) [27].

Three studies reported on the incidence of liver problems [6, 13, 15]. Two studies found no statistically significant differences between the screen-detected and clinically detected unscreened groups [6, 15]. In the remaining study, there were significantly fewer cases of hepatopathy in the screen detected group (1/9, median age 5.06 years) compared to the unscreened clinical group (4/5, median age 9.4 years, *p* = 0.02) [13].

### Visual problems

Six studies (from five cohorts) reported on eye problems [13, 21, 22, 24–26]. Five of the six did not find a significant difference between screen detected and clinically detected groups. In one study, retinopathy was significantly less common amongst screen detected individuals (3/9, 100%) than clinically detected after screening individuals (5/5, 100%), *p* = 0.03 [13].

### Motor and muscular problems

Four studies reported on motor and muscular problems [6, 21, 25, 26]. Three of these did not find a significant difference between screen detected and clinically detected groups. There were significantly fewer cases of hypotonia/myopathy in the screen detected group (4/10, 40%) compared to the clinically detected after screening group (14/17, 82.4%) in the remaining study, *p* = 0.03) [6].

### Hypoglycaemia

Two studies explored hypoglycaemia (not as a presenting symptom), and are presented in [6, 22]. One of the 2 studies found a significant difference between screen detected and clinically detected groups (*p* = 0.02) [6]. They found 4 out of 10 (40%) cases in the screen detected group compared to 15 out of 17 (88%) in the clinically detected after screening group.

### 3) asymptomatic screened, symptomatic screened, and late clinically detected patients

Four studies were included in this follow up analysis [6, 13, 25, 26] . Details are provided in Fig. 5 and Additional file 5.

No statistically significant differences between groups were found for mortality (1 study [6]),  liver problems (2 studies [13, 26]), neurological problems (1 study [26]), muscular/motor problems (1 study [26]) or hypoglycaemia (1 study [26]).

Differences in the incidence of heart problems were reported in two studies [13, 26]. There was a statistically significant difference between group and cardiomyopathy incidence in one study (*p* < 0.05) [13]. This was driven by a higher frequency of cardiomyopathy in the late clinically diagnosed group (5/5, median age 12.2 years) compared to the asymptomatic screened group (1/6, median age 3.2 years). There was no statistically significant difference in the incidence of arrhythmias between the three groups in the second study (*p* = 1) [26].

### Visual problems

Three studies reported visual problems across the 3 separate groups [13, 25, 26]. There was no significant difference in 2 studies. One study found a significant difference between the groups in terms of retinopathy: asymptomatic screening group = 1/6 (16.7%), symptomatic screening group = 1/3 (33.3%), late clinical detection group = 4/5 (80%), *p* = 0.05 [13]. No statistically significant differences were observed in pairwise comparison. This may be due to a lack of statistical power related to the small sample sizes.

## Discussion

We conducted a systematic review to examine the clinical outcomes of people with LCHADD/MTPD who received early dietary management following screening (universal newborn screening, cascade testing or incidental detection) versus later dietary management following the presentation of symptoms (either before or after the screening period). We included 13 articles, reporting on 11 patient groups. The methodological quality of all of the included studies was weak or moderate.

From our narrative synthesis there appear to be fewer instances of heart and liver related problems in people with LCHADD or MTPD who are diagnosed earlier (either through newborn screening, cascade testing, or incidental detection) than those diagnosed at a later age (following symptomatic presentation). However, it is not clear whether these differences are due to a beneficial effect of screening or biases in study design, and it is less clear whether there is any reduction in mortality following screen-detection. Mortality may occur earlier in those the early screen detected group. This may be due to these newborns being symptomatic at diagnosis and having a more severe form of the disease. 6/13 studies [6, 9, 15, 24, 28, 29] were concerned with the health benefits of treatment during life, and did not report on mortality. Therefore, we do not have a complete picture on whether the treatments in those studies would have had an effect on mortality. We undertook additional pre-specified follow up analyses to explore differences in outcomes between 1) asymptomatically vs symptomatically detected patients, (2) screened vs unscreened patients and (3) people who were asymptomatic at screening vs those who were symptomatic at screening vs those who were clinically detected in the absence of screening or who were clinically detected following false negative screening results. The majority of studies did not show a statistically significant difference between any of the groups across the three comparisons. Across follow up analyses 1 and 2 (comprising 58 individual comparisons in total), eight comparisons reached statistical significance across four studies [6, 13, 27, 29] (see Additional file 5 for details). For follow up analysis 3, only one (out of 10) 3-way comparisons performed was statistically significant when the group who presented with symptoms before screening took place were analysed separately, with greater instances of cardiomyopathy in the late clinical group than in the earlier asymptomatic screened group [13]. The limited number of statistically significant results in the follow up comparisons performed may be due to the low number of patients per comparison group (1–44 patients per comparison group, the vast majority of groups comprising less than 10 patients) resulting in low power to reject a false null hypothesis. There are also inherent biases in these analyses. Follow up analysis 1 comparing asymptomatic (detected by newborn screening, cascade testing or incidentally) cases versus cases with symptoms at diagnosis (early or late symptomatic) reflects the way the majority of the papers reported their data. This is the comparison of most interest in assessing the benefits of implementing screening. However, this comparison is biased towards screening as the most severe cases with symptoms before or at the time of newborn screening will all be allocated to the ‘symptomatic’ group. Follow up analysis 2 comparing screened (asymptomatic or early symptomatic) versus unscreened (early or late symptomatic) cases is less biased due to the allocation of the severe early presenting cases but is less applicable to the screening question as in current practice; babies with symptoms before or at the time of newborn screening would receive diagnostic testing anyway and would not benefit from screening. Age may have been a confounding factor in this follow up analysis as well. Those with more follow-up have more time in which an event can be recorded in the study, with events missed in those with insufficient follow up. Age at the time of the study was reported in eight out of the 10 studies [9, 13, 21–29]. In seven of those eight, the patients in the ‘screened’ group were considerably younger than the patients in the ‘unscreened’ groups (median ages of 2–10 years in the ‘screened’ groups compared to median ages between 19 and 22 years in the ‘unscreened’ groups [13, 21–29]. Most of the ‘unscreened’ group were cases ascertained prior to the introduction of universal newborn screening, so their disease may have progressed further than in the screen-detected cases. By doing follow up analysis 3, comparing symptomatic screened, asymptomatic screened and clinically detected patients (either unscreened or false negative screening test), we were able to reduce some of these biases, however, very few studies reported these data and sample sizes per group were very small.

A related review we undertook highlighted an issue in the indexing of some search terms in MEDLINE. The phrase “inborn errors of metabolism” had not been indexed as a MeSH heading, so further searches were done using this phrase as a key word search. Only one additional paper was identified that was relevant for this review.

Our systematic review has a number of strengths. To our knowledge, this is the first systematic review comparing the outcomes of LCHADD and MTPD patients following pre-symptomatic detection or clinical detection after presenting with symptoms. We conducted a wide-ranging exhaustive search with no limits on date or language, independent literature screening and quality appraisal were undertaken by two reviewers, and all data extraction forms were checked by a second reviewer. However, there are also some limitations. Although we did not exclude studies on the basis of language, the search terms were in English so may have missed papers in other languages.

Drawing conclusions from the follow up analyses should be interpreted with caution due to the small sample sizes. This review considers the *statistical* significance in the observed comparisons, but given the very few number of patients who have these disorders, it is important to also consider the *clinical* significance. Avoiding cardiomyopathy and hepatomegaly is of serious clinical importance to patients. On the other hand, enforcing a restricted diet on patients who have screened positive but who remain asymptomatic and may have never gone on to become symptomatic, may seriously affect their quality of life.

Evidence is still very limited and at risk of bias. Further investigation is needed regarding whether the cases of LCHADD and MTPD detected by screening represent the same spectrum of disease as those detected clinically, and whether all screen-detected babies would go on to become symptomatic. Confounding factors such as age at time of study, follow-up time, possible repetition of patients across cohorts, genotype and severity of disease must all be considered. Analyses of genotype phenotype correlations might be of assistance in the future. Likewise, large international collaborations may help provide a clearer picture on whether pre-symptomatic treatment results in better outcomes.

## Conclusions

There is some evidence to suggest that pre-symptomatic dietary management may help delay or prevent the onset of some LCHADD and MTPD related long-term complications. However, studies have not been large enough to show any consistent significant benefits, and many confounding factors such as genotype, disease severity, age at diagnosis, and follow-up time between ‘early’ and ‘late’ treated groups have not been accounted for. An internationally collaborative research effort is needed to fully examine the risks and the benefits to pre-emptive dietary management with particular attention being paid to disease severity and treatment group.

## Supplementary information

**Additional file 1.** Search strategies.

**Additional file 2.** Data extraction form for primary studies.

**Additional file 3.** List of excluded studies and reasons.

**Additional file 4.** Methodological quality of included studies.

**Additional file 5.** Follow up analyses.

## Data Availability

The datasets supporting the conclusions of this article are included within the article and its additional files.

## Abbreviations

DHA: Docosahexaenoic acid
EPHPP: Effective Public Health Practice Project
LCHAD: Long-chain 3-hydroxyacyl-CoA dehydrogenase
LCHADD: Long-chain 3-hydroxyacyl-CoA dehydrogenase deficiency
MCT: Medium-chain triglycerides
MTP/mTFP: Mitochondrial trifunctional protein
MTPD: Mitochondrial trifunctional protein deficiency
NBS: Newborn blood spot
NSC: National Screening Committee
PRISMA: Preferred Reporting Items for Systematic Reviews and Meta-Analyses
TFP: Trifunctional protein
TFPD: Mitochondrial trifunctional protein disorder
UK: United Kingdom

## References

[1] Moorthie S (2014). Systematic review and meta-analysis to estimate the birth prevalence of five inherited metabolic diseases. J Inherit Metab Dis.

[2] Wanders RJA (1990). Long-chain 3-HYDROXYACYL-COA dehydrogenase-deficiency - identification of a new inborn error of mitochondrial fatty-acid BETA-oxidation. J Inherit Metab Dis.

[3] Wanders R (1990). Long-chain 3-hydroxyacyl-CoA dehydrogenase deficiency: identification of a new inborn error of mitochondrial fatty acidβ-oxidation. J Inherit Metab Dis.

[4] Yang Z (2002). Fetal genotypes and pregnancy outcomes in 35 families with mitochondrial trifunctional protein mutations. Am J Obstet Gynecol.

[5] Spiekerkoetter U (2010). Current issues regarding treatment of mitochondrial fatty acid oxidation disorders. J Inherit Metab Dis.

[6] Spiekerkoetter U (2009). Management and outcome in 75 individuals with long-chain fatty acid oxidation defects: results from a workshop. J Inherit Metab Dis.

[7] Bo R (2017). Clinical and molecular investigation of 14 Japanese patients with complete TFP deficiency: a comparison with Caucasian cases. J Hum Genet.

[8] Sander J (2005). Neonatal screening for defects of the mitochondrial trifunctional protein. Mol Genet Metab.

[9] Sperk A, Mueller M, Spiekerkoetter U (2010). Outcome in six patients with mitochondrial trifunctional protein disorders identified by newborn screening. Mol Genet Metab.

[10] Boutron A., Acquaviva C., Vianey-Saban C., de Lonlay P., de Baulny H. Ogier, Guffon N., Dobbelaere D., Feillet F., Labarthe F., Lamireau D., Cano A., de Villemeur T. Billette, Munnich A., Saudubray J.M., Rabier D., Rigal O., Brivet M. (2011). Comprehensive cDNA study and quantitative analysis of mutant HADHA and HADHB transcripts in a French cohort of 52 patients with mitochondrial trifunctional protein deficiency. Molecular Genetics and Metabolism.

[11] den Boer ME (2003). Mitochondrial trifunctional protein deficiency: a severe fatty acid oxidation disorder with cardiac and neurologic involvement. J Pediatr.

[12] den Boer ME (2002). Long-chain 3-hydroxyacyl-CoA dehydrogenase deficiency: clinical presentation and follow-up of 50 patients. Pediatrics.

[13] Karall D (2015). Clinical outcome, biochemical and therapeutic follow-up in 14 Austrian patients with long-chain 3-Hydroxy acyl CoA dehydrogenase deficiency (LCHADD). Orphanet J Rare Dis.

[14] Lindner M (2011). Efficacy and outcome of expanded newborn screening for metabolic diseases-report of 10 years from south-West Germany. Orphanet J Rare Dis.

[15] Lund AM (2012). Biochemical screening of 504,049 newborns in Denmark, the Faroe Islands and Greenland--experience and development of a routine program for expanded newborn screening. Mol Genetics Metab.

[16] Frazier DM (2006). The tandem mass spectrometry newborn screening experience in North Carolina: 1997-2005. J Inherit Metab Dis.

[17] Zytkovicz TH (2001). Tandem mass spectrometric analysis for amino, organic, and fatty acid disorders in newborn dried blood spots: a two-year summary from the New England newborn screening program. Clin Chem.

[18] Thomas B (2004). A process for systematically reviewing the literature: providing the research evidence for public health nursing interventions. Worldviews Evid-Based Nurs.

[19] Effective Public Health Practice Project. Quality Assessment Tool For Quantitative Studies. Hamilton, ON: Effective Public Health Practice Project. 1998 20/06/2019]; Available from: https://merst.ca/wp-content/uploads/2018/02/quality-assessment-tool_2010.pdf

[20] Nyaga V.N., M. Arbyn, and M.J.A.o.P.H. Aerts, Metaprop: a Stata command to perform meta-analysis of binomial data. Archives of Public Health. 2014;72(1):39. 10.1186/2049-3258-72-39 PMC4373114 25810908

[21] Fahnehjelm KT (2008). Ocular characteristics in 10 children with long-chain 3-hydroxyacyl-CoA dehydrogenase deficiency: a cross-sectional study with long-term follow-up. Acta Ophthalmol.

[22] Fahnehjelm KT (2016). Most patients with long-chain 3-hydroxyacyl-CoA dehydrogenase deficiency develop pathological or subnormal retinal function. Acta Paediatr.

[23] Haglind CB (2013). Growth in long-chain 3-Hydroxyacyl-CoA dehydrogenase deficiency. Jimd Reports.

[24] Immonen T (2016). Earlier diagnosis and strict diets improve the survival rate and clinical course of long-chain 3-hydroxyacyl-CoA dehydrogenase deficiency. Acta Paediatr.

[25] Boese EA (2016). Characterization of Chorioretinopathy associated with mitochondrial Trifunctional protein disorders: long-term follow-up of 21 cases. Ophthalmology.

[26] De Biase I (2017). Diagnosis, treatment, and clinical outcome of patients with mitochondrial Trifunctional protein/long-chain 3-Hydroxy acyl-CoA dehydrogenase deficiency. Jimd Reports.

[27] Gillingham MB (2017). Triheptanoin versus trioctanoin for long-chain fatty acid oxidation disorders: a double blinded, randomized controlled trial. J Inherit Metab Dis.

[28] Kang E (2018). Clinical and genetic characteristics of patients with fatty acid oxidation disorders identified by newborn screening. BMC Pediatr.

[29] Sykut-Cegielska J (2011). Urgent metabolic service improves survival in long-chain 3-hydroxyacyl-CoA dehydrogenase (LCHAD) deficiency detected by symptomatic identification and pilot newborn screening. J Inherit Metab Dis.

