# Supplementary material for: Evaluation of earlier versus later dietary management in long-chain 3-hydroxyacyl-CoA dehydrogenase or mitochondrial trifunctional protein deficiency: a systematic review
Source: Orphanet J Rare Dis. 2019 Nov 15;14:258. doi: 10.1186/s13023-019-1226-y (PMC6858661; doi:10.1186/s13023-019-1226-y)
Supplement: Supplementary file 1 — Additional file 1. Search strategies. [file 13023_2019_1226_MOESM1_ESM.docx]

**Additional file 1**. Search strategies

Search strategies for MEDLINE, MEDLINE In-Process, MEDLINE Daily, Epub Ahead of Print, Embase, and the Cochrane Library before deduplication

|  | References retrieved | De-duplicated references |
| --- | --- | --- |
| Medline | 2977 | 2964 |
| PreMedline | 238 | 237 |
| Embase | 4597 | 3785 |
| Web of Science | 911 | 301 |
| Cochrane | 240 | 196 |
| **TOTAL** | **8963** | **7483** |

Search strategy for MEDLINE

| # | Search terms | Results |
| --- | --- | --- |
| 1 | (mitochondrial trifunctional protein adj3 deficien*).ti,ab,kf | 60 |
| 2 | mtp deficien*.ti,ab,kf. | 46 |
| 3 | trifunctional protein deficien*.ti,ab,kf. | 69 |
| 4 | (LCHAD or LCHADD).mp | 166 |
| 5 | Long-Chain-3-Hydroxyacyl-CoA Dehydrogenase/ | 97 |
| 6 | 3-Hydroxyacyl-CoA Dehydrogenase/df [Deficiency] | 2 |
| 7 | HADH Deficien*.mp. | 3 |
| 8 | (Hydroxacyl and dehydrogenase).mp. | 5 |
| 9 | long chain.mp. | 23373 |
| 10 | 8 and 9 | 1 |
| 11 | hydroxydicarboxylic acidur*.mp. | 18 |
| 12 | Lipid Metabolism, Inborn Errors/ | 2587 |
| 13 | fatty acid oxidation disorder*.mp. | 248 |
| 14 | Acyl-CoA Dehydrogenase/df [Deficiency] | 223 |
| 15 | 1 or 2 or 3 or 4 or 5 or 6 or 7 or 10 or 11 or 12 or 13 | 2977 |

Search strategy for PreMedline

| # | Search terms | Results |
| --- | --- | --- |
| 1 | (mitochondrial trifunctional protein adj3 deficien*).ti,ab,kf | 10 |
| 2 | mtp deficien*.ti,ab,kf. | 5 |
| 3 | trifunctional protein deficien*.ti,ab,kf. | 9 |
| 4 | (LCHAD or LCHADD).mp | 29 |
| 5 | Long-Chain-3-Hydroxyacyl-CoA Dehydrogenase/ | 18 |
| 6 | 3-Hydroxyacyl-CoA Dehydrogenase/df [Deficiency] | 45 |
| 7 | HADH Deficien*.mp. | 0 |
| 8 | (Hydroxacyl and dehydrogenase).mp. | 0 |
| 9 | long chain.mp. | 2950 |
| 10 | 8 and 9 | 0 |
| 11 | hydroxydicarboxylic acidur*.mp. | 0 |
| 12 | (Lipid Metabolism and Inborn Errors).mp | 1 |
| 13 | Acyl-CoA Dehydrogenase/df [Deficiency] | 159 |
| 14 | fatty acid oxidation disorder*.mp. | 41 |
| 15 | 1 or 2 or 3 or 4 or 5 or 6 or 7 or 10 or 11 or 12 or 13 | 238 |

Search strategy for Embase

| # | Search terms | Results |
| --- | --- | --- |
| 1 | (mitochondrial trifunctional protein adj3 deficien*).ti,ab,hw | 90 |
| 2 | mtp deficien*.ti,ab,hw | 84 |
| 3 | trifunctional protein deficien*.ti,ab,hw | 90 |
| 4 | (LCHAD or LCHADD).mp | 323 |
| 5 | HADH Deficien*.mp. | 4 |
| 6 | (Hydroxacyl and dehydrogenase).mp. | 9 |
| 7 | long chain.mp. | 34778 |
| 8 | 6 and 7 | 2 |
| 9 | hydroxydicarboxylic acidur*.mp. | 18 |
| 10 | exp 3 hydroxyacyl coenzyme A dehydrogenase/ | 1487 |
| 11 | long chain 3 hydroxyacyl coenzyme A dehydrogenase/ | 83 |
| 12 | acyl coenzyme A dehydrogenase/ | 1984 |
| 13 | exp long chain acyl coenzyme A dehydrogenase/ | 567 |
| 14 | ”inborn error of metabolism”/ | 12393 |
| 15 | lipid metabolism.mp. or lipid metabolism/ | 94904 |
| 16 | 14 and 15 | 167 |
| 17 | fatty acid oxidation disorder*.mp. | 471 |
| 18 | 1 or 2 or 3 or 4 or 5 or 8 or 9 or 10 or 11 or 12 or 13 or 16 or 17 | 4597 |

Search strategy for Web of Science

| # | Search terms | Results |
| --- | --- | --- |
| 1 | ((("fatty acid oxidation disorder*" OR "LCHADD" OR "LCHAD" OR "Long-Chain-3-Hydroxyacyl-CoA Dehydrogenase" OR "HADH deficien*" OR ("hydroxyacyl" AND "dehydrogenase" AND "long chain")))) OR TS=(("hydroxydicarboxylic acidur*" or ("inborn errors" and "lipid metabolism"))) OR TS=("mtp deficien*" or ("mitochondrial protein" NEAR/3 deficien*) or ("trifunctional protein" near/3 deficien*)) | 911 |

Search strategy for Cochrane Library

| # | Search terms | Results |
| --- | --- | --- |
| 1 | MTP deficien* | 5 |
| 2 | Trifunctional protein near/3 deficien* | 7 |
| 3 | lchad or lchadd | 9 |
| 4 | MeSH descriptor: [Long-Chain-3-Hydroxyacyl-CoA Dehydrogenase] explode all trees | 1 |
| 5 | MeSH descriptor: [3-Hydroxyacyl CoA Dehydrogenases] explode all trees | 86 |
| 6 | (Hydroxacyl and dehydrogenase) and long chain | 0 |
| 7 | Hydroxydicarboxylic acidur* | 1 |
| 8 | MeSH descriptor: [Acyl-CoA Dehydrogenase] explode all trees | 13 |
| 9 | MeSH descriptor [Acyl-CoA Dehydrogenase, Long-Chain] explode all trees | 2 |
| 10 | Mitochondrial trifunctional protein near/3 deficien* | 0 |
| 11 | MeSH descriptor: [Lipid Metabolism, Inborn Errors] this term only | 22 |
| 12 | Fatty acid oxidation disorder* | 121 |
| 13 | #1 or #2 or #3 or #4 or #5 or #6 or #7 or #8 or #9 or #10 or#11 or #12 | 240 |
