# Supplementary material for: Evaluation of earlier versus later dietary management in long-chain 3-hydroxyacyl-CoA dehydrogenase or mitochondrial trifunctional protein deficiency: a systematic review
Source: Orphanet J Rare Dis. 2019 Nov 15;14:258. doi: 10.1186/s13023-019-1226-y (PMC6858661; doi:10.1186/s13023-019-1226-y)
Supplement: Supplementary file 2 — Additional file 2. Data extraction form for primary studies. [file 13023_2019_1226_MOESM2_ESM.doc]

**Data extraction form for primary studies**

*Name of first reviewer: Name of second reviewer:*

| **Study details** | | | | |
| --- | --- | --- | --- | --- |
| Study ID (Endnote ref) | |  | | |
| First author surname and year of publication | |  | | |
| Country | |  | | |
| Study design | |  | | |
| Study setting | |  | | |
| Number of centres | |  | | |
| Time period / study duration | |  | | |
| Follow up period | |  | | |
| Funding | |  | | |
| Competing interests | |  | | |
| **Review question** | | | | |
| Prevalence (1)  Genotype-phenotype (2)  Screening test (3)  Early vs late treament (4) |  | | | |
| **Aim of the study** | | | | |
|  | | | | |
| **Description of study format (study design/set up)** | | | | |
|  | | | | |
| **Patient selection** | | | | |
| Inclusion criteria: | | | | |
| Exclusion criteria:  NR | | | | |
|  | | | | |
| **Study flow** | | | | |
| **Item** | | **All** | **early treated** | **Late treated** |
| Screened | |  |  |  |
| Randomised/Included in study | |  |  |  |
| Excluded from study (reasons) | |  |  |  |
| Included in analysis | |  |  |  |
| Excluded from analysis (reasons) | |  |  |  |

| **Baseline characteristics** | | | |
| --- | --- | --- | --- |
| **Item** | **All** | **Screened** | **Clinical** |
| Total number at baseline |  |  |  |
| Age mean (SD)  Median (range)  [at last follow up] |  |  |  |
| Male n (%) |  |  |  |
| Female n (%) |  |  |  |
| LCHADD n (%)  1528G>C gene n (%) |  |  |  |
| MTP n (%) |  |  |  |
| Ethnicity |  |  |  |
| Way of diagnosis |  |  |  |
| Age at diagnosis, median (range) |  |  |  |
| Age at the start of dietary management, median (range) |  |  |  |
| Affected family members (y/n) |  |  |  |
| Comments on how late has been defined  Late defined as anyone found clinically | | | |

| **Interventions & comparators** | | | | |
| --- | --- | --- | --- | --- |
| **1) Prevalence** | | | | |
|  | **Screened cohort** | | **Unscreened cohort** | **Historic cohort** |
| Screening programme in operation? (y/n) |  | |  |  |
| Confirmation of disease |  | |  |  |
| Follow-up (years) |  | |  |  |
| **2) Genotype/phenotype** |  | |  |  |
| **Mutation type** | **Homozygous 1528G>C**  (isolated LCHADD) | | **Compound heterozygous** (LCHADD) | **LCHADD/**  **MTP**  (specify mutations) |
| Number (%) |  | |  |  |
| **3) TMS screening method** | | | | |
| Source and type of material | |  | | |
| Age at specimens collection | |  | | |
| Time between taking the sample and freezing it | |  | | |
| Samples pooled? | |  | | |
| Method of extraction & TMS analysis | |  | | |
| Type of tandem MS | |  | | |
| Data management | |  | | |
| Quality assurance | |  | | |
| Analyte(s) | |  | | |
| Cut-off / Threshold | |  | | |
| Cut-off prespecified (y/n) | |  | | |
| Positive screening results | |  | | |
| Reference standard used | |  | | |
| Follow-up (years) | |  | | |
| Number received index test, n (%) | |  | | |
| Number received reference standard n (%) | |  | | |
| Time between index test and reference standard | |  | | |
| Uptake (%) | |  | | |
| **4) Dietary management** | | | | |
|  | **NBS** | | **Late - symptomatic** |  |
| Total number |  | |  |  |
| Way of diagnosis |  | |  |  |
| Age at start of dietary management mean (median) |  | |  |  |
| Type of dietary management  Low-fat diet n (%)  MCT n (%)  Triheptanoin n (%)  Essential fatty acids n (%)  DHA n (%)  Carnitine n (%)  Cornstarch n (%) |  | |  |  |
| Other dietary management or comments on dietary management: | | | | |

| **Outcomes** | | | | |
| --- | --- | --- | --- | --- |
| **1)Prevalence of LCHADD/MTP** | | | | |
| Reported outcomes: NA | | | | |
|  | **Screened cohort** | | **Unscreened cohort** | **Historic cohort** |
| Number screened |  | |  |  |
| Number of identified cases |  | |  |  |
| Birth prevalence |  | |  |  |
| Cases per 100,000 |  | |  |  |
| Incidence of asymptomatic cases |  | |  |  |
| Notes/comments: NA | | | | |
| **2) Genotype/phenotype** | | | | |
| **Mutation type** | **Homozygous 1528G>C**  (isolated LCHADD) | | **Compound heterozygous** (LCHADD) | **LCHADD/**  **MTP**  (specify mutations) |
| **Severity**  Neonatal severe n (%)  Infant hepatic n (%)  Late onset neuromyopathic n (%) |  | |  |  |
| **Maternal phenotype**  AFLP n (%)  HELLP n (%)  Normal n (%) |  | |  |  |
| **Death**  n (%) |  | |  |  |
| **Age at time of death** (months) |  | |  |  |
| Other outcomes |  | |  |  |
|  | | | | |
| **3) Screening for LCHADD/MTP** | | | | |
| Reported outcomes: | | | | |
| Total number screened | |  | | |
| TP | |  | | |
| TN | |  | | |
| FP | |  | | |
| FN | |  | | |
| Sensitivity, % (95% CI) | |  | | |
| Specificity, % (95% CI) | |  | | |
| PPV, % (95% CI) | |  | | |
| NPV, % (95% CI) | |  | | |
| Other conditions detected by test | |  | | |
| Other (specify) | |  | | |
| Notes / Comments: | | | | |
| **4) Dietary treatment** | | | | |
| Delete those NA | **Screened** | | **Clinical** |  |
| **Number included patients n (%)** |  | |  |  |
| **Death n (%)** |  | |  |  |
| **Age at death mean (range)** |  | |  |  |
| Others (specify): |  | |  |  |
| Notes/comments: | | | | |

| **Authors’ comments & conclusion** |
| --- |
|  |
| **Reviewer’s comments & conclusion** |
|  |
