# Supplementary material for: Evaluation of earlier versus later dietary management in long-chain 3-hydroxyacyl-CoA dehydrogenase or mitochondrial trifunctional protein deficiency: a systematic review
Source: Orphanet J Rare Dis. 2019 Nov 15;14:258. doi: 10.1186/s13023-019-1226-y (PMC6858661; doi:10.1186/s13023-019-1226-y)
Supplement: Supplementary file 3 — Additional file 3. List of excluded studies and reasons. [file 13023_2019_1226_MOESM3_ESM.docx]

Additional file 3: Publications excluded after review of full-text articles

| Reference | Reason for exclusion |
| --- | --- |
| Adriaenssens, K. Van Sande, M. 1968, [Detection of metabolic diseases], Acta Neurologica et Psychiatrica Belgica | Review |
| Albasanz Gallan, J. L. [Genetics and metabolic disorders], Medicina Tropical | Review |
| Aleck, K. Partup, A. Shub, M. Harrison, H. Roe, C. 1997, Acute fatty liver of pregnancy (AFLP) is associated with fetal long chain 3-hydroxyacyl COA dehydrogenase (LCHAD) deficiency, American Journal of Human Genetics | Abstract only |
| Alonso, J. B. Gomez, R. G. Nieto, J. S. Martin, M. G. Lopez, V. M. N. Manso, G. M. Salinas, C. S. Cardona, A. L. U. 2015, Innate errors of metabolism in a pediatric hospital. Substantial differences between the extended neonatal pre- and post screening era. [Spanish], Revista Espanola de Pediatria | No LCHADD cases |
| Angdisen, J. Moore, V. D. Cline, J. M. Payne, R. M. Ibdah, J. A. 2005, Mitochondrial trifunctional protein defects: molecular basis and novel therapeutic approaches, Current Drug Targets - Immune Endocrine & Metabolic Disorders | Review |
| Arya, R. Candelier, C. K. 2001, Neonatal long chain 3-hydroxyacyl-coenzyme A dehydrogenase deficiency (LCHAD) presenting as liver disease in the mother, Journal of Obstetrics and Gynaecology | Case study only 1 case |
| Autti-Ramo, I. Makela, M. Sintonen, H. Koskinen, H. Laajalahti, L. Halila, R. Kaariainen, H. Lapatto, R. Nanto-Salonen, K. Pulkki, K. Renlund, M. Salo, M. Tyni, T. 2005, Expanding screening for rare metabolic disease in the newborn: an analysis of costs, effect and ethical consequences for decision-making in Finland, Acta Paediatrica | Not relevant |
| Babiker, O. Flanagan, S. E. Ellard, S. Girim, H. A. Hussain, K. Senniappan, S. 2015, Protein-induced hyperinsulinaemic hypoglycaemia due to a homozygous HADH mutation in three siblings of a Saudi family, Journal of Pediatric Endocrinology and Metabolism | Not LCHADD |
| Baily, M. A. Becker Jr, W. Hayes, M. Clayton, E. W. 2005, Exploring options for expanded newborn screening, Journal of Law, Medicine and Ethics | Review |
| Barnerias, C. Vianey-Saban, C. Brivet, M. Rabier, D. Touati, G. De Lonlay, P. Saudubray, J. M. 2005, TWO CASES OF PERIPHERAL NEUROPATHY REVEALING TRIFUNCTIONAL PROTEIN DEFICIENCY, Journal of Inherited Metabolic Disease | Abstract only |
| Bartlett, K. Eaton, S. J. Pourfarzam, M. 1997, New developments in neonatal screening, Archives of Disease in Childhood: Fetal and Neonatal Edition | Review |
| Bartoshesky, L. E. 2003, Newborn screening in Delaware, Delaware Medical Journal | Review |
| Baruteau, J. Sachs, P. Broue, P. Brivet, M. Abdoul, H. Vianey-Saban, C. Ogier de Baulny, H. 2013, Clinical and biological features at diagnosis in mitochondrial fatty acid beta-oxidation defects: a French pediatric study of 187 patients, Journal of Inherited Metabolic Disease | No information by patient |
| Baumgartner, C. Baumgartner, D. 2006, Biomarker discovery, disease classification, and similarity query processing on high-throughput MS/MS data of inborn errors of metabolism, Journal of Biomolecular Screening | High risk population |
| Behrend, A. M. Harding, C. O. Shoemaker, J. D. Matern, D. Sahn, D. J. Elliot, D. L. Gillingham, M. B. 2012, Substrate oxidation and cardiac performance during exercise in disorders of long chain fatty acid oxidation, Molecular Genetics & Metabolism | Not relevant |
| Bergmann, S. R. Herrero, P. Sciacca, R. Hartman, J. J. Rubin, P. J. Hickey, K. T. Epstein, S. Kelly, D. P. 2001, Characterization of altered myocardial fatty acid metabolism in patients with inherited cardiomyopathy, Journal of Inherited Metabolic Disease | No age or method of diagnosis only current age and outcomes |
| Berry, S. A. Jurek, A. M. Anderson, C. Bentler, K. Region 4 Genetics Collaborative Priority, Workgroup, 2010, The inborn errors of metabolism information system: A project of the Region 4 Genetics Collaborative Priority 2 Workgroup, Genetics in Medicine | Not relevant |
| Bessey, A. Chilcott, J. Pandor, A. Paisley, S. 2014, The Cost-Effectiveness of Expanding the Nhs Newborn Bloodspot Screening Programme To Include Homocystinuria (Hcu), Maple Syrup Urine Disease (Msud), Glutaric Aciduria Type 1 (Ga1), Isovaleric Acidaemia (Iva), and Long-Chain Hydroxyacyl-Coa Dehydrogenase Deficiency (Lchadd), Value in Health | abstract only |
| Bieneck, H. C. Ask, S. Halldin, M. Gardman, J. Nyberg, G. Alm, J. von Dobeln, U. Nordenstrom, A. 2008, Growth in 10 Swedish patients with long-chain 3OH-Acyl-CoA dehydrogenase (LCHAD) deficiency, Journal of Inherited Metabolic Disease | abstract only |
| Bieneck, H. C. Nordenstrom, A. Halldin, M. Alm, J. Nemeth, A. Ask, S. Nyberg, G. Holmstrom, G. Tear, F. K. von Dobeln, U. 2007, Clinical follow-up of 10 children with long-chain 30H-acyl-CoA dehydrogenase (LCHAD) deficiency, Journal of Inherited Metabolic Disease | abstract only |
| Boles, R. G. Buck, E. A. Blitzer, M. G. Platt, M. S. Gowan, T. M. Martin, S. K. Yoon, H. R. Madsen, J. A.  Reyes-Mugica, M. Rinaldo, P. (1998). "Retrospective biochemical screening of fatty acid oxidation disorders in postmorterm livers of 418 cases of sudden death in the first year of life." Journal of Pediatrics 132(6): 924-933. | No groups |
| Bonnet, D. Martin, D. Pascale De, Lonlay; Villain, E; Jouvet, P; Rabier, D; Brivet, M; Saudubray, J. M;  1999, "Arrhythmias and conduction defects as presenting symptoms of fatty acid oxidation disorders in children." Circulation 100(22): 2248-2253. | no age or method of diagnosis |
| Bonnet, D. Martin, D. PoggiTravert, F. Villain, E. Kachaner, J. Acar, P. Saudubray, J. M. 1997,  "Arrhythmias and conduction defects as a presenting symptom of fatty-acid oxidation disorders in children." Circulation 96(8): 2437-2437. | abstract only |
| Chakrapani, A. Olpin, S. Cleary, M. Walter, J. H. Wraith, J. E. Besley, G. T. (2000). "Trifunctional protein deficiency: three families with significant maternal hepatic dysfunction in pregnancy not associated with E474Q mutation." Journal of Inherited Metabolic Disease 23(8): 826-834. | No screened group |
| Chen, C. H. Chi, C. C. Shu, N. Y. 1991, Recurrent acute encephalopathy due to fatty acid metabolic defect--report of two cases, Zhonghua yi xue za zhi = Chinese medical journal; Free China ed | Both incidental cases |
| Chrastina, P. Kostalova, E. Paulova, M. Varholakova, L. Stastna, S. Elleder, M. Zeman, J. (2008). "LCHAD deficiency - The most frequent fatty acid oxidation disorder in newborn screening in the Czech Republic." Journal of Inherited Metabolic Disease 31: 29-29. | abstract only |
| Cipriano, L. E. Rupar, C. A. Zaric, (2007) The cost-effectiveness of expanding newborn screening for up to 21 inherited metabolic disorders using tandem mass spectrometry: results from a decision-analytic model (Structured abstract). Value in Health 10, 83-97 | Review |
| Ciske, J. B. Hoffman, G. Hanson, K. Annable, K. M. Wolff, J. Litsheim, T. Laessig, R. Aronson, R. (2000). "Newborn screening in Wisconsin: program overview and test addition." WMJ 99(2): 38-42. | Not relevant |
| Coates, P. M. (1998). "Fatty acid metabolism in mitochondria: defects and genetics." Biofactors 7(3): 201-202. | Review |
| Crocker, A. C. (1976). "Inborn errors of lipid metabolism: early identification." Clinics in Perinatology 3(1): 99-113. | Review |
| Crouch, W. H., Jr. and C. M. Evanhoe (1967). "Inborn errors of metabolism." Pediatric Clinics of North America 14(1): 269-282. | Review |
| den Boer, M. E. Dionisi-Vici, C. Chakrapani, A. van Thuijl, A. O. Wanders, R. J. Wijburg, F. A. (2003). "Mitochondrial trifunctional protein deficiency: a severe fatty acid oxidation disorder with cardiac and neurologic involvement." Journal of Pediatrics 142(6): 684-689. | don't have outcome or age at diagnosis and outcome, just severity |
| den Boer, M. E. Ijlst, L. Wijburg, F. A. Oostheim, W. van Werkhoven, M. A. van Pampus, M. G. Heymans, H. S. Wanders, R. J. (2000). "Heterozygosity for the common LCHAD mutation (1528g>C) is not a major cause of HELLP syndrome and the prevalence of the mutation in the Dutch population is low." Pediatric Research 48(2): 151-154. | no outcome data |
| den Boer, M. E. Wanders, R. J. Morris, A. A. I. Jlst L; Heymans, H. S. Wijburg, F. A. (2002). "Long-chain 3-hydroxyacyl-CoA dehydrogenase deficiency: clinical presentation and follow-up of 50 patients." Pediatrics 109(1): 99-104. | patients aren't grouped by age of diagnosis |
| Dereddy, N. R. Kronn, D. Krishnan, U. Dereddy, N. R., et al. (2009). "Defects in long chain fatty acid oxidation presenting as severe cardiomyopathy and cardiogenic shock in infancy." Cardiology in the Young 19(5): 540-542. | no screened vs unscreened group |
| Ding, J. H. Yang, B. Z. Nada, M. A. Roe, C. R. (1996). "Long-chain 3-hydroxyacyl-CoA dehydrogenase deficiency: The major disease-causing mutation and diagnosis." Pediatric Research 39(4): 851-851. | No early group |
| Dionisivici, C. Garavaglia, B. Burlina, A. Bertini, E. Wanders, R. J. A. Hashimoto, T. Sabetta, G. Invernizzi, F. Taroni, F. Didonato, S. 1995, "MITOCHONDRIAL TRIFUNCTIONAL PROTEIN-DEFICIENCY - CLINICAL AND BIOCHEMICAL FINDINGS IN POOR PATIENTS." Annals of Neurology 38(3): 544-544. | No early group |
| Divry, P. Vianey-Saban, C. Mathieu, M. (1999). "Determination of total fatty acids in plasma: cis-5-tetradecenoic acid (C14:1 omega-9) in the diagnosis of long-chain fatty acid oxidation defects." Journal of Inherited Metabolic Disease 22(3): 286-288. | no age at diagnosis data |
| Djouadi, F. Habarou, F. Le Bachelier, C. Ferdinandusse, S. Schlemmer, D. Benoist, J. F. Boutron, A. Andresen, B. S. Visser, G. de Lonlay, P. Olpin, S. Fukao, T. Yamaguchi, S. Strauss, A. W. Wanders, R. J. Bastin, J. (2016). "Mitochondrial trifunctional protein deficiency in human cultured fibroblasts: effects of bezafibrate." Journal of Inherited Metabolic Disease 39(1): 47-58. | Not relevant |
| Dogan, E. Uysal, S. Ozturk, Y. Arslan, N. (2017). "Selective screening for inborn errors of metabolism: A report of six years experience." Iranian Journal of Pediatrics 27 (5) (no pagination)(e11323). | Not relevant |
| Domingo, S. J. L. Koninckx, C. R. Serra, J. D. Calvete, J. F. Tomas, M. L. C. Gomez, A. G. Rubio, A. (1995). "Long-chain acyl coenzyme A dehydrogenase deficiency: A new case, DEFIENCIA DE ACIL COA DESHIDROGENASA DE CADENA LARGA. CASO CLINICO." Anales Espanoles de Pediatria 42(6): 456-458. | single case |
| Duran, M. Deklerk, J. B. C. Pollthe, B. T. Wanders, R. J. A. Huymans, J. G. M. (1991). "LONG-CHAIN 3-HYDROXYACYL-COA DEHYDROGENASE-DEFICIENCY - PLASMA AND URINE ORGANIC-ACIDS." American Journal of Human Genetics 49(4): 53-53. | all diagnosed through cascade testing. |
| Estrella, J. Wilcken, B. Carpenter, K. Bhattacharya, K. Tchan, M. Wiley, V. (2014). "Expanded newborn screening in New South Wales: missed cases." Journal of Inherited Metabolic Disease 37(6): 881-887. | Not relevant |
| Fincke, M. L. (1965). "Inborn Errors of Metabolism." Journal of the American Dietetic Association 46: 280-284. | review |
| Finsterer, J. and S. Zarrouk-Mahjoub (2017). "Trifunctional Protein Deficiency Due to HADHB Mutations Is a Multisystem, beta-Oxidation Disorder." Archives of Iranian Medicine 20(12): 767-769. | letter |
| Francis, D. E. (1979). "Inborn errors of metabolism: the need for sugar." Journal of Human Nutrition 33(2): 146-154. | Abstract only |
| Francois, J. (1975). "Ocular manifestations of inborn errors of carbohydrate and lipid metabolism." Bibliotheca Ophthalmologica: Supplementa ad Ophthalmologica(84): I-VII, 1-175. | review |
| Fukushima, K., et al. (2004). "Lack of common mutation in the alfa-subunit of the mitochondrial trifunctional protein and the polymorphism of CYP2E1 in three Japanese women with acute fatty liver of pregnancy/HELLP syndrome." Hepatology Research 30(4): 226-231. | Not relevant |
| Gillingham, M. (2006) Nutritional therapy and clinical outcomes in children with LCHAD deficiency. Inborn error review series (dietary management of inborn errors) 16, 6 | no full text available |
| Gillingham, M. Van Calcar, S. Ney, D. Wolff, J. Harding, C. (1999). "Dietary management of long-chain 3-hydroxyacyl-CoA dehydrogenase deficiency (LCHADD). A case report and survey." Journal of Inherited Metabolic Disease 22(2): 123-131. | single case |
| Gillingham, M. B. Connor, W. E. Matern, D. Rinaldo, P. Burlingame, T. Meeuws, K. Harding, C. O.Gillingham, M. B., et al. (2003). "Optimal dietary therapy of long-chain 3-hydroxyacyl-CoA dehydrogenase deficiency." Molecular Genetics & Metabolism 79(2): 114-123. | treatment only. No mapping of cases and outcomes just dosage |
| Gillingham, M. B., et al. (2015). "Odd or even? Results from a randomized trial of triheptanoin compared to MCT in patients with long-chain fatty acid oxidation disorders." Molecular Genetics and Metabolism 114(3): 320-321. | Abstract |
| GILLINGHAM, M. B., JORDAN, J., STADLER, D. & HARDING, C. O. 2005. Effects of increased dietary protein on energy balance and metabolic control in children with long-chain 3-hydroxy acyl-CoA dehydrogenase (LCHAD) deficiency. Molecular Genetics and Metabolism, 84, 220-220. | Abstract |
| GILLINGHAM, M. B., MATERN, D. & HARDING, C. O. 2009. Effect of Feeding, Exercise and Genotype on Plasma 3-Hydroxyacylcarnitines in Children with Lchad Deficiency. Topics in Clinical Nutrition, 24, 359-365. | No outcomes |
| GILLINGHAM, M. B., PURNELL, J. Q., JORDAN, J., STADLER, D., HAQQ, A. M. & HARDING, C. O. 2007. Effects of higher dietary protein intake on energy balance and metabolic control in children with long-chain 3-hydroxy acyl-CoA dehydrogenase (LCHAD) or trifunctional protein (TFP) deficiency. Molecular Genetics & Metabolism, 90, 64-9. | No outcomes |
| GILLINGHAM, M. B., SCOTT, B., ELLIOTT, D. & HARDING, C. O. 2006. Metabolic control during exercise with and without medium-chain triglycerides (MCT) in children with long-chain 3-hydroxy acyl-CoA dehydrogenase (LCHAD) or trifunctional protein (TFP) deficiency. Molecular Genetics & Metabolism, 89, 58-63. | No outcomes |
| GLASGOW, J. F. T., MOORE, R., ROBINSON, P. H. & MCKIERNAN, P. J. 1992. The phenylpropionic acid load test: Experience with 72 children at-risk for beta-oxidation disorders. Irish Journal of Medical Science, 161, 586-588. | Not relevant |
| GOKMEN-OZEL, H., DALY, A., DAVIES, P., CHAHAL, S. & MACDONALD, A. 2010. Errors in emergency feeds in inherited metabolic disorders: A randomised controlled trial of three preparation methods. Archives of Disease in Childhood, 95, 776-780. | Not relevant |
| GOLBAHAR, J., AL-JISHI, E. A., ALTAYAB, D. D., CARREON, E., BAKHIET, M. & ALKHAYYAT, H. 2013. Selective newborn screening of inborn errors of amino acids, organic acids and fatty acids metabolism in the Kingdom of Bahrain. Molecular Genetics & Metabolism, 110, 98-101. | Not relevant |
| GREENBERG, C. R., MHANNI, A. A., CORKERY, T., SALTEL-OLSON, J., MALLORY, C. & SEARGEANT, L. 2007. Whole blood palmitate oxidation as a screening test for fatty acid oxidation disorders: A five-year experience. Journal of Inherited Metabolic Disease, 30, 47-47. | Abstract |
| GREGERSEN, N., ANDRESEN, B. S. & BROSS, P. 2000. Prevalent mutations in fatty acid oxidation disorders: diagnostic considerations. European Journal of Pediatrics, 159 Suppl 3, S213-8. | Review |
| GREGERSEN, N., ANDRESEN, B. S., CORYDON, M. J., CORYDON, T. J., OLSEN, R. K., BOLUND, L. & BROSS, P. 2001. Mutation analysis in mitochondrial fatty acid oxidation defects: Exemplified by acyl-CoA dehydrogenase deficiencies, with special focus on genotype-phenotype relationship. Human Mutation, 18, 169-89. | Review |
| GREGERSEN, N. & OLSEN, R. K. 2010. Disease mechanisms and protein structures in fatty acid oxidation defects. Journal of Inherited Metabolic Disease, 33, 547-53. | Review |
| GU, X. F., HAN, L. S., GAO, X. L., YAN, Y. L., YE, J. & QIU, W. J. 2004. A pilot study of selective screening for high risk children with inborn error of metabolism using tandem mass spectrometry in China. [Chinese]. *Zhonghua er ke za zhi,* Chinese journal of pediatrics. 42, 401-404. | Not relevant |
| HAGENBUCHNER, J., SCHOLL-BUERGI, S., KARALL, D. & AUSSERLECHNER, M. J. 2018. Very long-/ and long Chain-3-Hydroxy Acyl CoA Dehydrogenase Deficiency correlates with deregulation of the mitochondrial fusion/fission machinery. *Scientific Reports,* 8, 3254. | Cell study |
| HAGENFELDT, L., VENIZELOS, N. & VON DOBELN, U. 1995. Clinical and biochemical presentation of long-chain 3-hydroxyacyl-CoA dehydrogenase deficiency. *Journal of Inherited Metabolic Disease,* 18, 245-8. | Review |
| HAGLIND, C. B., NORDENSTROM, A., ASK, S., VON DOBELN, U., GUSTAFSSON, J. & STENLID, M. H. 2015. Erratum to: increased and early lipolysis in children with long-chain 3-hydroxyacyl-CoA dehydrogenase (LCHAD) deficiency during fast.[Erratum for J Inherit Metab Dis. 2015 Mar;38(2):315-22; PMID: 25141826]. *Journal of Inherited Metabolic Disease,* 38, 377. | Erratum |
| HAGLIND, C. B., NORDENSTROM, A., ASK, S., VON DOBELN, U., GUSTAFSSON, J. & STENLID, M. H. 2015. Increased and early lipolysis in children with long-chain 3-hydroxyacyl-CoA dehydrogenase (LCHAD) deficiency during fast. *Journal of Inherited Metabolic Disease,* 38, 315-322. | Not relevant |
| HAGLIND, C. B., STENLID, M. H., ASK, S., ALM, J., NEMETH, A., DOBELN, U. & NORDENSTROM, A. 2013. Growth in Long-Chain 3-Hydroxyacyl-CoA Dehydrogenase Deficiency. *Jimd Reports,* 8, 81-90. | No late group |
| HALE, D. E., BATSHAW, M. L. & COATES, P. M. 1985. Long-chain acyl coenzyme A dehydrogenase deficiency: An inherited cause of nonketotic hypoglycemia. *Pediatric Research,* 19, 666-671. | Review |
| HALE, D. E., STANLEY, C. A. & COATES, P. M. 1990. Genetic defects of acyl-CoA dehydrogenases: studies using an electron transfer flavoprotein reduction assay. *Progress in clinical and biological research,* 321, 333-348. | Review |
| HALE, D. E., STANLEY, C. A. & COATES, P. M. 1990. The long-chain acyl-CoA dehydrogenase deficiency. *Progress in clinical and biological research,* 321, 303-311. | Review |
| HALE, D. E., THORPE, C., BRAAT, K., WRIGHT, J. H., ROE, C. R., COATES, P. M., HASHIMOTO, T. & GLASGOW, A. M. 1990. The L-3-hydroxyacyl-CoA dehydrogenase deficiency. *Progress in clinical and biological research,* 321, 503-510. | Single case |
| HALL, P. L., MARQUARDT, G., MCHUGH, D. M. S., CURRIER, R. J., TANG, H., STOWAY, S. D. & RINALDO, P. 2014. Postanalytical tools improve performance of newborn screening by tandem mass spectrometry. *Genetics in Medicine,* 16, 889-895. | Not LCHADD |
| HAN, L., HAN, F., YE, J., QIU, W., ZHANG, H., GAO, X., WANG, Y., JI, W. & GU, X. 2015. Spectrum analysis of common inherited metabolic diseases in Chinese patients screened and diagnosed by tandem mass spectrometry. *Journal of Clinical Laboratory Analysis,* 29, 162-8. | Not LCHADD |
| HAN, L. S., YE, J., QIU, W. J., GAO, X. L., WANG, Y. & GU, X. F. 2007. Selective screening for inborn errors of metabolism on clinical patients using tandem mass spectrometry in China: a four-year report. *Journal of Inherited Metabolic Disease,* 30, 507-14. | Not LCHADD |
| HAN, L. S., YE, J., QIU, W. J., GAO, X. L., WANG, Y., ZHANG, Y. J. & GU, X. F. 2007. Application of tandem mass spectrometry on the diagnosis of fatty acid oxidation disorders. [Chinese]. *Chinese Journal of Medical Genetics,* 24, 692-695. | Not relevant |
| HARDING, C. O., GILLINGHAM, M. B., VAN CALCAR, S. C., WOLFF, J. A., VERHOEVE, J. N. & MILLS, M. D. 1999. Docosahexaenoic acid and retinal function in children with long-chain 3-hydroxyacyl-CoA dehydrogenase deficiency. *Journal of Inherited Metabolic Disease,* 22, 276-80. | No outcome data |
| HARDING, C. O., SCOTT, B. & GILLINGHAM, M. B. 2005. MCT supplementation immediately prior to exercise improves exercise tolerance in children with long-chain 3-hydroxy acyl-CoA dehydrogenase (LCHAD) deficiency. *Molecular Genetics and Metabolism,* 84, 222-222. | Abstract only |
| HARZER, K. 1979. [Prenatal diagnosis of incurable familial metabolic diseases. Prenatal diagnosis of disorders of lipid metabolism]. *Medizinische Welt,* 30, 1810-6. | Review |
| HE, M., PEI, Z., MOHSEN, A. W., WATKINS, P., MURDOCH, G., VAN VELDHOVEN, P. P., ENSENAUER, R. & VOCKLEY, J. 2011. Identification and characterization of new long chain Acyl-CoA dehydrogenases. *Molecular Genetics and Metabolism,* 102, 418-429. | Not relevant |
| HEALTH QUALITY, O. 2003. Neonatal screening of inborn errors of metabolism using tandem mass spectrometry: an evidence-based analysis. *Ontario Health Technology Assessment Series,* 3, 1-36. | Review and not LCHADD |
| HINTON, C. F., MAI, C. T., NABUKERA, S. K., BOTTO, L. D., FEUCHTBAUM, L., ROMITTI, P. A., WANG, Y., PIPER, K. N. & OLNEY, R. S. 2014. Developing a public health-tracking system for follow-up of newborn screening metabolic conditions: a four-state pilot project structure and initial findings. *Genetics in Medicine,* 16, 484-90. | Not relevant |
| HOFFMANN, G. F., VON KRIES, R., KLOSE, D., LINDNER, M., SCHULZE, A., MUNTAU, A. C., ROSCHINGER, W., LIEBL, B., MAYATEPEK, E. & ROSCHER, A. A. 2004. Frequencies of inherited organic acidurias and disorders of mitochondrial fatty acid transport and oxidation in Germany. *European Journal of Pediatrics,* 163, 76-80. | Not LCHADD |
| HUSSA, C., FICICIOGLU, C., VERONA, M., GANESH, J., PAYAN, I., PATANO, J., LIEBHART, R. & YUDKOFF, M. 2006. Is breastfeeding an option in the dietary management of long chain fatty acid oxidation disorders? Our experience with seven patients. *Journal of Inherited Metabolic Disease,* 29, 113-113. | Abstract |
| IBARRA-GONZALEZ, I., FERNANDEZ-LAINEZ, C., BELMONT-MARTINEZ, L., GUILLEN-LOPEZ, S., MONROY-SANTOYO, S. & VELA-AMIEVA, M. 2014. Characterization of inborn errors of intermediary metabolism in mexican patients. [Spanish]. *Anales de Pediatria,* 80, 310-316. | No information on diagnosis |
| IBDAH, I., SIMS, H., GIBSON, B., PIZZURRO, M., TREEM, W., BENNETT, M. & STRAUSS, A. 1996. The molecular basis of long chain 3-hydroxyacyl-CoA dehydrogenase (LCHAD) deficiency. *Faseb Journal,* 10, 2195-2195. | Abstract |
| IBDAH, J. A., ISAACS, J., TREEM, W., BENNETT, M. & STRAUSS, A. W. 1996. The molecular basis of acute fatty liver of pregnancy associated with pediatric long chain 3-hydroxyacyl-CoA dehydrogenase deficiency. *Hepatology,* 24, 300-300. | Abstract |
| IBDAH, J. A., ISAACS, J., TREEM, W. & STRAUSS, A. W. 1996. Acute fatty liver of pregnancy and maternal long chain 3-hydroxyacyl-CoA dehydrogenase. *Gastroenterology,* 110, A1215-A1215. | Abstract |
| IBDAH, J. A., TEIN, I., DIONISI-VICI, C., BENNETT, M. J., L, I. J., GIBSON, B., WANDERS, R. J. & STRAUSS, A. W. 1998. Mild trifunctional protein deficiency is associated with progressive neuropathy and myopathy and suggests a novel genotype-phenotype correlation. *Journal of Clinical Investigation,* 102, 1193-9. | No early or late group information |
| IBDAH, J. A., YANG, Z. & BENNETT, M. J. 2000. Liver disease in pregnancy and fetal fatty acid oxidation defects. *Molecular Genetics & Metabolism,* 71, 182-9. | Review |
| IJLST, L., OOSTHEIM, W., RUITER, J. P. N. & WANDERS, R. J. A. 1997. Molecular basis of long-chain 3-hydroxyacyl-CoA dehydrogenase deficiency: Identification of two new mutations. *Journal of Inherited Metabolic Disease,* 20, 420-422. | No early or late group information |
| IJLST, L., RUITER, J. P. N., VREIJLING, J. & WANDERS, R. J. A. 1996. Long-chain 3-hydroxyacyl-CoA dehydrogenase deficiency: A new method to identify the G1528C mutation in genomic DNA showing its high frequency ( 90%) and identification of a new mutation (T2198C). *Journal of Inherited Metabolic Disease,* 19, 165-168. | Single case |
| IJLST, L., USKIKUBO, S., KAMIJO, T., HASHIMOTO, T., RUITER, J. P., DE KLERK, J. B. & WANDERS, R. J. 1995. Long-chain 3-hydroxyacyl-CoA dehydrogenase deficiency: high frequency of the G1528C mutation with no apparent correlation with the clinical phenotype. *Journal of Inherited Metabolic Disease,* 18, 241-4. | No early or late group information |
| INSINGA, R. P., LAESSIG, R. H. & HOFFMAN, G. L. 2002. Newborn screening with tandem mass spectrometry: examining its cost-effectiveness in the Wisconsin newborn screening panel (Structured abstract). *Journal of Pediatrics* [Online], 141. Available: http://cochranelibrary-wiley.com/o/cochrane/cleed/articles/NHSEED-22002001875/frame.html | Not LCHADD |
| ITO, T. 2015. Mass Screening for Inborn Errors of Metabolism. [Japanese]. *Rinsho byori,* The Japanese journal of clinical pathology. 63, 441-449. | Review |
| IWANCZAK, F. & SMIGIEL, R. 2004. The most common genetic inherited defects of the protein and fat metabolism in children. [Polish]. *Gastroenterologia Polska,* 11, 375-383. | Review |
| JACKSON, S., BARTLETT, K., LAND, J., MOXON, E. R., POLLITT, R. J., LEONARD, J. V. & TURNBULL, D. M. 1991. Long-chain 3-hydroxyacyl-CoA dehydrogenase deficiency. *Pediatric Research,* 29, 406-411. | no screened cases |
| JIANG, M., LIU, L., MEI, H., LI, X., CHENG, J. & CAI, Y. 2015. Detection of inborn errors of metabolism using GC-MS: Over 3 years of experience in southern China. *Journal of Pediatric Endocrinology and Metabolism,* 28, 375-380. | Not relevant |
| JOHNSON, D. W. & TRINH, M. U. 2003. Analysis of isomeric long-chain hydroxy fatty acids by tandem mass spectrometry: application to the diagnosis of long-chain 3-hydroxyacyl CoA dehydrogenase deficiency. *Rapid Communications in Mass Spectrometry,* 17, 171-5. | Not relevant |
| KAMIJO, T., WANDERS, R. J., SAUDUBRAY, J. M., AOYAMA, T., KOMIYAMA, A. & HASHIMOTO, T. 1994. Mitochondrial trifunctional protein deficiency. Catalytic heterogeneity of the mutant enzyme in two patients. *Journal of Clinical Investigation,* 93, 1740-7. | Animal study |
| KAUR, M. & BIRLA, S. 2008. Threshold challenge in India-newborn screening for aminoacids, organic acids and fatty acid oxidation disorders. *Journal of Inherited Metabolic Disease,* 31, 150-150. | Abstract |
| KELLY, D. P., MENDELSOHN, N. J., SOBEL, B. E. & BERGMANN, S. R. 1993. Detection and assessment by positron emission tomography of a genetically determined defect in myocardial fatty acid utilization (long-chain acyl-CoA dehydrogenase deficiency). *American Journal of Cardiology,* 71, 738-744. | All late diagnosed |
| KEPPEN, L. D. & RANDALL, B. 1999. Inborn defects of fatty acid oxidation: a preventable cause of SIDS. *South Dakota journal of medicine,* 52, 187-188; disscussion 188-189. | Single case and not LCHADD |
| KIMURA, M. & YAMAGUCHI, S. 2001. Trifunctional protein deficiency and long-chain-3-hydroxy-acyl CoA dehydrogenase deficiency. [Japanese]. *Ryoikibetsu shokogun shirizu*, 77-79. | No LCHADD |
| KLOSE, D. A., KOLKER, S., HEINRICH, B., PRIETSCH, V., MAYATEPEK, E., VON KRIES, R. & HOFFMANN, G. F. 2002. Incidence and short-term outcome of children with symptomatic presentation of organic acid and fatty acid oxidation disorders in Germany. *Pediatrics,* 110, 1204-11. | Don’t know age or method of diagnosis for LCHADD cases |
| KOBAYASHI, H., HASEGAWA, Y., ENDO, M., PUREVSUREN, J. & YAMAGUCHI, S. 2007. A retrospective ESI-MS/MS analysis of newborn blood spots from 18 symptomatic patients with organic acid and fatty acid oxidation disorders diagnosed either in infancy or in childhood. *Journal of Inherited Metabolic Disease,* 30, 606. | Abstract |
| KONG, X. F., ZHANG, X. X., YU, Y. Y., SHI, Q., LA, D. D., ZHU-GE, C. D., DENG, L., GONG, Q. M., SHEN, B. Y., PENG, C. H. & LI, H. W. 2007. No mutation was found in the alpha-subunit of the mitochondrial tri-functional protein in one patient with severe acute fatty liver of pregnancy and her relatives. *Journal of Gastroenterology & Hepatology,* 22, 2107-11. | 1 case |
| KORENKE, G. C., MARQUARDT, I., MOTZ, R., VOGES, A., WANDERS, R. J. A., STEUERWALD, U. & SANDER, J. 2005. Long-chain hydroxyacyl-CoA dehydrogenase deficiency-LCHAD defect. Two-year follow-up of two patients. [German]. *Monatsschrift fur Kinderheilkunde,* 153, 657-663. | Both cases screened |
| L, I. J., RUITER, J. P., HOOVERS, J. M., JAKOBS, M. E. & WANDERS, R. J. 1996. Common missense mutation G1528C in long-chain 3-hydroxyacyl-CoA dehydrogenase deficiency. Characterization and expression of the mutant protein, mutation analysis on genomic DNA and chromosomal localization of the mitochondrial trifunctional protein alpha subunit gene. *Journal of Clinical Investigation,* 98, 1028-33. | Yeast cell study |
| L, I. J., WANDERS, R. J., USHIKUBO, S., KAMIJO, T. & HASHIMOTO, T. 1994. Molecular basis of long-chain 3-hydroxyacyl-CoA dehydrogenase deficiency: identification of the major disease-causing mutation in the alpha-subunit of the mitochondrial trifunctional protein. *Biochimica et Biophysica Acta,* 1215, 347-50. | No early vs late groups |
| LABARTHE, F. 2008. New therapeutic approaches in mitochondrial fatty acid oxidation disorders. [French]. *Archives de Pediatrie,* 15, 608-610. | Review |
| LABARTHE, F., BENOIST, J. F., BRIVET, M., VIANEY-SABAN, C., DESPERT, F. & DE BAULNY, H. O. 2005. PARTIAL HYPOPARATHYROIDISM ASSOCIATED WITH A MITOCHONDRIAL TRIFUNCTIONAL PROTEIN DEFICIENCY. *Journal of Inherited Metabolic Disease,* 28, 105-105. | Abstract |
| LANDAU, Y. E., LICHTER-KONECKI, U. & LEVY, H. L. 2014. Genomics in newborn screening. *Journal of Pediatrics,* 164, 14-19. | Review |
| LANDAU, Y. E., WAISBREN, S. E., CHAN, L. M. & LEVY, H. L. 2017. Long-term outcome of expanded newborn screening at Boston children's hospital: benefits and challenges in defining true disease. *Journal of Inherited Metabolic Disease,* 40, 209-218. | No outcomes |
| LANTHALER, B., WIESER, S., DEUTSCHMANN, A., SCHOSSIG, A., FAUTH, C., ZSCHOCKE, J. & WITSCH-BAUMGARTNER, M. 2014. Genotype-based databases for variants causing rare diseases. *Gene,* 550, 136-40. | Not LCHADD |
| LAW, E. L. K. 2010. Diagnosis of fatty acid oxidation disorders by mass spectrometry. *Clinica Chimica Acta,* 411, 906-906. | Abstract |
| LI, F., YANG, Z., ZHANG, A., SUN, X., WANG, J. & MENG, R. 2015. [The changes of LCHAD in preeclampsia with different clinical features and the correlation with NADPH P47-phox, p38MAPK-alpha, COX-2 and serum FFA and TG]. *Chung-Hua Fu Chan Ko Tsa Chih [Chinese Journal of Obstetrics & Gynecology],* 50, 92-100. | No outcomes |
| LINDNER, M., HOFFMANN, G. F. & MATERN, D. 2010. Newborn screening for disorders of fatty-acid oxidation: experience and recommendations from an expert meeting. *Journal of Inherited Metabolic Disease,* 33, 521-6. | Not relevant |
| LOEBER, J. G. 2007. Neonatal screening in Europe; the situation in 2004.[Erratum appears in J Inherit Metab Dis. 2008 Jun;31(3):469]. *Journal of Inherited Metabolic Disease,* 30, 430-8. | Review and no LCHADD |
| LUCAS, T. G., HENRIQUES, B. J., RODRIGUES, J. V., BROSS, P., GREGERSEN, N. & GOMES, C. M. 2011. Cofactors and metabolites as potential stabilizers of mitochondrial acyl-CoA dehydrogenases. *Biochimica et Biophysica Acta - Molecular Basis of Disease,* 1812, 1658-1663. | Not relevant |
| LUKACS, Z. 2009. Newborn screening in Germany, Austria and Switzerland : CCCurrent status. [German]. *Monatsschrift fur Kinderheilkunde,* 157, 1209-1214. | Review |
| LUND, A. M., DIXON, M. A., VREKEN, P., LEONARD, J. V. & MORRIS, A. A. M. 2003. What is the role of medium-chain triglycerides in the management of long-chain 3-hydroxyacyl-CoA dehydrogenase deficiency? *Journal of Inherited Metabolic Disease,* 26, 353-360. | Not relevant |
| LUND, A. M. & LEONARD, J. V. 2001. Feeding difficulties in long-chain 3-hydroxyacyl-CoA dehydrogenase deficiency. *Archives of Disease in Childhood,* 85, 487-488. | Not relevant |
| LUND, A. M., SKOVBY, F., VESTERGAARD, H., CHRISTENSEN, M. & CHRISTENSEN, E. 2010. Clinical and biochemical monitoring of patients with fatty acid oxidation disorders. *Journal of Inherited Metabolic Disease,* 33, 495-500. | Review |
| LUNDEMOSE, J. B., KOLVRAA, S., GREGERSEN, N., CHRISTENSEN, E. & GREGERSEN, M. 1997. Fatty acid oxidation disorders as primary cause of sudden and unexpected death in infants and young children: an investigation performed on cultured fibroblasts from 79 children who died aged between 0-4 years. *Molecular Pathology,* 50, 212-7. | Not LCHADD |
| MACDONALD, A., WEBSTER, R., WHITLOCK, M., GERRARD, A., DALY, A., PREECE, M. A., EVANS, S., ASHMORE, C., CHAKRAPANI, A., VIJAY, S. & SANTRA, S. 2018. The safety of Lipistart, a medium-chain triglyceride based formula, in the dietary treatment of long-chain fatty acid disorders: a phase I study. *Journal of Pediatric Endocrinology & Metabolism,* 31, 297-304. | 1 case |
| MALVAGIA, S., HAYNES, C. A., GRISOTTO, L., OMBRONE, D., FUNGHINI, S., MORETTI, E., MCGREEVY, K. S., BIGGERI, A., GUERRINI, R., YAHYAOUI, R., GARG, U., SEETERLIN, M., CHACE, D., DE JESUS, V. R. & LA MARCA, G. 2015. Heptadecanoylcarnitine (C17) a novel candidate biomarker for newborn screening of propionic and methylmalonic acidemias. *Clinica Chimica Acta,* 450, 342-8. | Not relevant |
| MARIN-GARCIA, J. & GOLDENTHAL, M. J. 2002. Fatty acid metabolism in cardiac failure: Biochemical, genetic and cellular analysis. *Cardiovascular Research,* 54, 516-527. | Review |
| MARQUARDT, G., CURRIER, R., MCHUGH, D. M., GAVRILOV, D., MAGERA, M. J., MATERN, D., OGLESBEE, D., RAYMOND, K., RINALDO, P., SMITH, E. H., TORTORELLI, S., TURGEON, C. T., LOREY, F., WILCKEN, B., WILEY, V., GREED, L. C., LEWIS, B., BOEMER, F., SCHOOS, R., MARIE, S., VINCENT, M. F., SICA, Y. C., DOMINGOS, M. T., AL-THIHLI, K., SINCLAIR, G., AL-DIRBASHI, O. Y., CHAKRABORTY, P., DYMERSKI, M., PORTER, C., MANNING, A., SEASHORE, M. R., QUESADA, J., REUBEN, A., CHRASTINA, P., HORNIK, P., ATEF MANDOUR, I., ATTY SHARAF, S. A., BODAMER, O., DY, B., TORRES, J., ZORI, R., CHEILLAN, D., VIANEY-SABAN, C., LUDVIGSON, D., STEMBRIDGE, A., BONHAM, J., DOWNING, M., DOTSIKAS, Y., LOUKAS, Y. L., PAPAKONSTANTINOU, V., ZACHARIOUDAKIS, G. S., BARATH, A., KARG, E., FRANZSON, L., JONSSON, J. J., BREEN, N. N., LESKO, B. G., BERBERICH, S. L., TURNER, K., RUOPPOLO, M., SCOLAMIERO, E., ANTONOZZI, I., CARDUCCI, C., CARUSO, U., CASSANELLO, M., LA MARCA, G., PASQUINI, E., DI GANGI, I. M., GIORDANO, G., CAMILOT, M., TEOFOLI, F., MANOS, S. M., PETERSON, C. K., MAYFIELD GIBSON, S. K., SEVIER, D. W., LEE, S. Y., PARK, H. D., KHNEISSER, I., BROWNING, P., GULAMALI-MAJID, F., WATSON, M. S., EATON, R. B., SAHAI, I., RUIZ, C., TORRES, R., SEETERLIN, M. A., STANLEY, E. L., HIETALA, A., MCCANN, M., CAMPBELL, C., HOPKINS, P. V., DE SAIN-VAN DER VELDEN, M. G., ELVERS, B., MORRISSEY, M. A., SUNNY, S., KNOLL, D., WEBSTER, D., FRAZIER, D. M., MCCLURE, J. D., SESSER, D. E., et al. 2012. Enhanced interpretation of newborn screening results without analyte cutoff values. *Genetics in Medicine,* 14, 648-55. | Not relevant |
| MARSDEN, D. 2003. Expanded newborn screening by tandem mass spectrometry: the Massachusetts and New England experience. *Southeast Asian Journal of Tropical Medicine & Public Health,* 34 Suppl 3, 111-4. | 1 case |
| MARTINEZ-QUINTANA, E., PENA-QUINTANA, L., ARTILES-VIZCAINO, J. A. & RODRIGUEZ-GONZALEZ, F. 2009. Long-chain 3-hydroxyacyl-coenzyme A dehydrogenase deficiency and cardiogenic shock. *International Journal of Cardiology,* 136, e1-2. | Letter |
| MARTINS, E., COSTA, A., SILVA, E., MEDINA, M., CARDOSO, M. L., VIANEY-SABAN, C., DIVRY, P. & VILARINHO, L. 1996. Lethal dilated cardiomyopathy due to long-chain 3-hydroxyacyl-CoA dehydrogenase deficiency. *Journal of Inherited Metabolic Disease,* 19, 373-374. | 1 case |
| MATERN, D., CUTHBERT, C. D., TORTORELLI, S., CHACE, D. H., HAHN, S. & RINALDO, P. 2004. The diagnosis of long-chain 3-hydroxyacyl-CoA dehydrogenase (LCHAD) deficiency by newborn screening using tandem mass spectrometry. *Pediatric Research,* 55, 272A-272A. | Abstract |
| MATERN, D., STRAUSS, A. W., HILLMAN, S. L., MAYATEPEK, E., MILLINGTON, D. S. & TREFZ, F. K. 1999. Diagnosis of mitochondrial trifunctional protein deficiency in a blood spot from the newborn screening card by tandem mass spectrometry and DNA analysis. *Pediatric Research,* 46, 45-9. | Genotype but before 2000 |
| MATSUBARA, Y. 1995. Hepatic mitochondrial fatty acid oxidation disorders. [Japanese]. *Ryoikibetsu shokogun shirizu*, 312-316. | Review |
| MATTHEWS, R. P., RUSSO, P., BERRY, G. T., PICCOLI, D. A. & RAND, E. B. 2002. Biliary atresia associated with a fatty acid oxidation defect. *Journal of Pediatric Gastroenterology and Nutrition,* 35, 624-628. | One case |
| MCCOIN, C. S., PICCOLO, B. D., KNOTTS, T. A., MATERN, D., VOCKLEY, J., GILLINGHAM, M. B. & ADAMS, S. H. 2016. Unique plasma metabolomic signatures of individuals with inherited disorders of long-chain fatty acid oxidation. *Journal of Inherited Metabolic Disease,* 39, 399-408. | Don’t know age or method of diagnosis |
| MCHUGH, D. M. S., CAMERON, C. A., ABDENUR, J. E., ABDULRAHMAN, M., ADAIR, O., AL NUAIMI, S. A., AHLMAN, H., ALLEN, J. J., ANTONOZZI, I., ARCHER, S., AU, S., AURAY-BLAIS, C., BAKER, M., BAMFORTH, F., BECKMANN, K., PINO, G. B., BERBERICH, S. L., BINARD, R., BOEMER, F., BONHAM, J., BREEN, N. N., BRYANT, S. C., CAGGANA, M., CALDWELL, S. G., CAMILOT, M., CAMPBELL, C., CARDUCCI, C., CARIAPPA, R., CARLISLE, C., CARUSO, U., CASSANELLO, M., CASTILLA, A. M., RAMOS, D. E. C., CHAKRABORTY, P., CHANDRASEKAR, R., RAMOS, A. C., CHEILLAN, D., CHIEN, Y. H., CHILDS, T. A., CHRASTINA, P., SICA, Y. C., COCHO DE JUAN, J. A., COLANDRE, M. E., ESPINOZA, V. C., CORSO, G., CURRIER, R., CYR, D., CZUCZY, N., D'APOLITO, O., DAVIS, T., DE SAIN-VAN DER VELDEN, M. G., PECELLIN, C. D., DI GANGI, I. M., DI STEFANO, C. M., DOTSIKAS, Y., DOWNING, M., DOWNS, S. M., DY, B., DYMERSKI, M., RUEDA, I., ELVERS, B., EATON, R., ECKERD, B. M., EL MOUGY, F., EROH, S., ESPADA, M., EVANS, C., FAWBUSH, S., FIJOLEK, K. F., FISHER, L., FRANZSON, L., FRAZIER, D. M., GARCIA, L. R. C., BERMEJO, M. S. G. V., GAVRILOV, D., GERACE, R., GIORDANO, G., IRAZABAL, Y. G., GREED, L. C., GRIER, R., GRYCKI, E., GU, X., GULAMALI-MAJID, F., HAGAR, A. F., HAN, L., HANNON, W. H., HASLIP, C., HASSAN, F. A., HE, M., HIETALA, A., HIMSTEDT, L., HOFFMAN, G. L., HOFFMAN, W., HOGGATT, P., HOPKINS, P. V., HOUGAARD, D. M., HUGHES, K., HUNT, P. R., HWU, W. L., HYNES, J., et al. 2011. Clinical validation of cutoff target ranges in newborn screening of metabolic disorders by tandem mass spectrometry: A worldwide collaborative project. *Genetics in Medicine,* 13, 230-254. | Not relevant |
| MERRIMAN, R. B., BACON, B. R., BRUNT, E. M., TETRI, B. A., PETERS, M. G. & STRAUSS, A. W. 2001. A common mutation of the long-chain hydroxyacyl-Co-A dehydrogenase enzyme of mitochondrial fatty acid beta-oxidation is not associated with non-alcoholic steatohepatitis. *Gastroenterology,* 120, A543-A543. | Abstract |
| MILLINGTON, D. S., TERADA, N., CHACE, D. H., CHEN, Y. T., DING, J. H., KODO, N. & ROE, C. R. 1992. The role of tandem mass spectrometry in the diagnosis of fatty acid oxidation disorders. *Progress in Clinical & Biological Research,* 375, 339-54. | Not relevant |
| MOORE, S. J., HAITES, N. E., BROOM, I., WHITE, I., COLEMAN, R. J., POURFARZAM, M. & MORRIS, A. A. M. 1998. Acylcarnitine analysis in the investigation of myopathy. *Journal of Inherited Metabolic Disease,* 21, 427-428. | Single case |
| MOORTHIE, S., CAMERON, L., SAGOO, G. S., BONHAM, J. R. & BURTON, H. 2014. Systematic review and meta-analysis to estimate the birth prevalence of five inherited metabolic diseases. *Journal of Inherited Metabolic Disease,* 37, 889-98. | Review |
| MOZRZYMAS, R., KONIKOWSKA, K. & REGULSKA-ILOW, B. 2017. Energy exchangers with LCT as a precision method for diet control in LCHADD. *Advances in Clinical & Experimental Medicine,* 26, 515-525. | Not relevant |
| MUELLER, P., SCHULZE, A., SCHINDLER, I., ETHOFER, T., BUEHRDEL, P. & CEGLAREK, U. 2003. Validation of an ESI-MS/MS screening method for acylcarnitine profiling in urine specimens of neonates, children, adolescents and adults. *Clinica Chimica Acta,* 327, 47-57. | Not relevant |
| MUTZE, S., AHILLEN, I., RUDNIK-SCHOENEBORN, S., EGGERMANN, T., LEENERS, B., NEUMAIER-WAGNER, P. M., KUSE, S., RATH, W. & ZERRES, K. 2007. Neither maternal nor fetal mutation (E474Q) in the alpha-subunit of the trifunctional protein is frequent in pregnancies complicated by HELLP syndrome. *Journal of Perinatal Medicine,* 35, 76-8. | No outcomes or follow up |
| NADLER, H. L. 1972. Allotransplantation for the treatment of inborn errors of metabolism. *Annals of Internal Medicine,* 77, 314-6. | Review |
| NAGARAJA, D., MAMATHA, S. N., DE, T. & CHRISTOPHER, R. 2010. Screening for inborn errors of metabolism using automated electrospray tandem mass spectrometry: study in high-risk Indian population. *Clinical Biochemistry,* 43, 581-8. | Not relevant |
| NAIKI, M., OCHI, N., KATO, Y. S., PUREVSUREN, J., YAMADA, K., KIMURA, R., FUKUSHI, D., HARA, S., YAMADA, Y., KUMAGAI, T., YAMAGUCHI, S. & WAKAMATSU, N. 2014. Mutations in HADHB, which encodes the beta-subunit of mitochondrial trifunctional protein, cause infantile onset hypoparathyroidism and peripheral polyneuropathy. *American Journal of Medical Genetics. Part A,* 164A, 1180-7. | Not LCHADD |
| NEDOSZYTKO, B., SIEMINSKA, A., STRAPAGIEL, D., DABROWSKI, S., SLOMKA, M., SOBALSKA-KWAPIS, M., MARCINIAK, B., WIERZBA, J., SKOKOWSKI, J., FIJALKOWSKI, M., NOWICKI, R. & KALINOWSKI, L. 2017. High prevalence of carriers of variant c.1528G>C of HADHA gene causing long-chain 3-hydroxyacyl-CoA dehydrogenase deficiency (LCHADD) in the population of adult Kashubians from North Poland. *PLoS ONE [Electronic Resource],* 12, e0187365. | No cases |
| OEY, N. A., DEN BOER, M. E., WIJBURG, F. A., VEKEMANS, M., AUGE, J., STEINER, C., WANDERS, R. J., WATERHAM, H. R., RUITER, J. P. & ATTIE-BITACH, T. 2005. Long-chain fatty acid oxidation during early human development. *Pediatric Research,* 57, 755-9. | Not relevant |
| OLPIN, S. E. 2013. Pathophysiology of fatty acid oxidation disorders and resultant phenotypic variability. *Journal of Inherited Metabolic Disease,* 36, 645-58. | Review |
| OLPIN, S. E., WEBB, J. M., CLARK, S., DALLEY, J., HIND, H., CROFT, J., COLYER, S., MANNING, N., SCOTT, C., KIRK, R., BONHAM, J., DOWLING, M., YAP, S., GLAMUZINA, E. & SHARRARD, M. 2015. Fatty acid oxidation flux data from 304 symptomatic patients diagnosed with a range of fatty acid oxidation disorders facilitates the prediction of phenotype in screen positive babies from Newborn Screening programs. *Journal of Inherited Metabolic Disease,* 1), S187. | Abstract |
| ORII, K. E., AOYAMA, T., WAKUI, K., FUKUSHIMA, Y., MIYAJIMA, H., YAMAGUCHI, S., ORII, T., KONDO, N. & HASHIMOTO, T. 1997. Genomic and mutational analysis of the mitochondrial trifunctional protein beta-subunit (HADHB) gene in patients with trifunctional protein deficiency. *Human Molecular Genetics,* 6, 1215-24. | Not relevant |
| OUNAP, K., KAHRE, T., ROOMETS, E., ZORDANIA, R., LAHT, T. M. & WANDERS, R. J. A. 2005. SCREENING FOR THE COMMON LCHAD MUTATION G1528C IN ESTONIAN POPULATION. *Journal of Inherited Metabolic Disease,* 28, 104-104. | Abstract |
| OURA, T. 1969. [Inborn errors of metabolism, associated with ocular symptoms]. *Nippon Ganka Kiyo - Folia Ophthalmologica Japonica - Bulletin of Japanese Ophthalmology,* 20, 749-58. | Review |
| OURA, T. & KOZAKI, M. 1969. [Congenital metabolic disorders with eye manifestations]. *Ganka - Ophthalmology,* 11, 872-83. | Review |
| OZASA, H. & TANAKA, K. 1988. Short chain and long chain acyl-CoA dehydrogenase deficiencies. [Japanese]. *Tanpakushitsu kakusan koso,* Protein, nucleic acid, enzyme. 33, 564-567. | Not relevant |
| PANDOR, A., EASTHAM, J., CHILCOTT, J., PAISLEY, S. & BEVERLEY, C. 2006. Economics of tandem mass spectrometry screening of neonatal inherited disorders. *International Journal of Technology Assessment in Health Care,* 22, 321-6. | Review |
| PARINI, R., GARAVAGLIA, B., SAUDUBRAY, J. M., BARDELLI, P., MELOTTI, D., ZECCA, G. & DI DONATO, S. 1991. Clinical diagnosis of long-chain acyl-coenzyme A-dehydrogenase deficiency: use of stress and fat-loading tests. *Journal of Pediatrics,* 119, 77-80. | Single case |
| PENG, M., LIU, L., JIANG, M., LIANG, C., ZHAO, X., CAI, Y., SHENG, H., OU, Z. & LUO, H. 2013. Measurement of free carnitine and acylcarnitines in plasma by HILIC-ESI-MS/MS without derivatization. *Journal of Chromatography B: Analytical Technologies in the Biomedical & Life Sciences,* 932, 12-8. | Not relevant |
| PICHLER, K., MICHEL, M., ZLAMY, M., SCHOLL-BUERGI, S., RALSER, E., JORG-STRELLER, M. & KARALL, D. 2017. Breast milk feeding in infants with inherited metabolic disorders other than phenylketonuria - a 10-year single-center experience. *Journal of Perinatal Medicine,* 45, 375-382. | No late cases |
| PIEKUTOWSKA-ABRAMCZUK, D., OLSEN, R. K., WIERZBA, J., POPOWSKA, E., JURKIEWICZ, D., CIARA, E., OLTARZEWSKI, M., GRADOWSKA, W., SYKUT-CEGIELSKA, J., KRAJEWSKA-WALASEK, M., ANDRESEN, B. S., GREGERSEN, N. & PRONICKA, E. 2010. A comprehensive HADHA c.1528G>C frequency study reveals high prevalence of long-chain 3-hydroxyacyl-CoA dehydrogenase deficiency in Poland. *Journal of Inherited Metabolic Disease,* 33 Suppl 3, S373-7. | No early vs late groups |
| POLLITT, R. J. 1993. Neonatal screening. *Journal of Clinical Pathology,* 46, 497-499. | Review |
| POLLITT, R. J. 1995. Disorders of mitochondrial long-chain fatty acid oxidation. *Journal of Inherited Metabolic Disease,* 18, 473-90. | Review |
| PONS, R., ROIG, M., RIUDOR, E., RIBES, A., BRIONES, P., ORTIGOSA, L., BALDELLOU, A., GIL-GIBERNAU, J., OLESTI, M., NAVARRO, C. & WANDERS, R. J. A. 1996. The clinical spectrum of long-chain 3-hydroxyacyl-CoA dehydrogenase deficiency. *Pediatric Neurology,* 14, 236-243. | All early diagnosed |
| POTTER, B. K., LITTLE, J., CHAKRABORTY, P., KRONICK, J. B., EVANS, J., FREI, J., SUTHERLAND, S. C., WILSON, K. & WILSON, B. J. 2012. Variability in the clinical management of fatty acid oxidation disorders: results of a survey of Canadian metabolic physicians. *Journal of Inherited Metabolic Disease,* 35, 115-23. | Not relevant |
| POWELL, C. K., ISAACS, J. D., SIMS, H. F. & STRAUSS, A. W. 1995. MOLECULAR CHARACTERIZATION OF FETAL LONG-CHAIN 3-HYDROXYACYL-COA DEHYDROGENASE-DEFICIENCY ASSOCIATED WITH ACUTE FATTY LIVER OF PREGNANCY. *Pediatric Research,* 37, A151-A151. | Abstract |
| POWELL, C. K., SIMS, H. F., BRACKETT, J. C. & STRAUSS, A. W. 1994. COMPLETE CHARACTERIZATION OF THE HUMAN CARDIAC LONG-CHAIN 3-HYDROXYACYL-COA DEHYDROGENASE CDNA AND GENE. *Circulation,* 90, 245-245. | Abstract |
| PRIMASSIN, S. & SPIEKERKOETTER, U. 2010. ESI-MS/MS measurement of free carnitine and its precursor gamma-butyrobetaine in plasma and dried blood spots from patients with organic acidurias and fatty acid oxidation disorders. *Molecular Genetics & Metabolism,* 101, 141-5. | No outcome data |
| PRZYREMBEL, H., JAKOBS, C., L, I. J., DE KLERK, J. B. C. & WANDERS, R. J. A. 1991. Long-chain 3-Hydroxyacyl-CoA dehydrogenase deficiency. *Journal of Inherited Metabolic Disease,* 14, 674-680. | 1 case |
| QUINTANA, E. M., QUINTANA, L. P. & GONZALEZ, F. R. 2007. Long-chain 3-hydroxyacyl-coenzyme a dehydrogenase deficiency and cardiomyopathy. *Revista Espanola De Cardiologia,* 60, 1332-1334. | Letter |
| REY, J. 1972. [Hereditary digestive enzyme defects]. *Medecine et Chirurgie Digestives,* 1, 41-4 contd. | Review |
| RICE, G. M. & STEINER, R. D. 2016. Inborn errors of metabolism (metabolic disorders). *Pediatrics in Review,* 37, 3-17. | Review |
| ROE, C. R. & BRUNENGRABER, H. 2015. Anaplerotic treatment of long-chain fat oxidation disorders with triheptanoin: Review of 15 years Experience. *Molecular Genetics & Metabolism,* 116, 260-8. | Don’t know method of diagnosis |
| ROE, C. R., ROE, D. S., WALLACE, M. & GARRITSON, B. 2007. Choice of oils for essential fat supplements can enhance production of abnormal metabolites in fat oxidation disorders. *Molecular Genetics & Metabolism,* 92, 346-50. | Cell study |
| ROOMETS, E., KIVELA, T. & TYNI, T. 2013. Early dietary therapy in preventing progression of retinopathy in long-chain 3-hydroxyacyl-CoA dehydrogenase (LCHAD) deficiency caused by the homozygous G1528C mutation. *Acta Ophthalmologica,* 91. | Abstract |
| SAKAKIDA, H. 1972. [Clinical practice of diet therapy in various diseases. 4. Abnormal metabolic regulation and its therapy by diet. (2) Lipid metabolism disorders and others]. *Kangogaku Zasshi - Japanese Journal of Nursing,* 36, 1056-9. | Review |
| SAUDUBRAY, J. M., MARTIN, D., DE LONLAY, P., TOUATI, G., POGGI-TRAVERT, F., BONNET, D., JOUVET, P., BOUTRON, M., SLAMA, A., VIANEY-SABAN, C., BONNEFONT, J. P., RABIER, D., KAMOUN, P. & BRIVET, M. 1999. Recognition and management of fatty acid oxidation defects: a series of 107 patients. *Journal of Inherited Metabolic Disease,* 22, 488-502. | No outcome data |
| SAUDUBRAY, J. M., MARTIN, D., POGGI-TRAVERT, F., BILLETTE DE VILLEMEUR, T., SPADA, M., BARTULI, A., JOUVET, P., BRIVET, M., SLAMA, A., VIANEY-LIAUD, C., DEMAUGRE, F., BONNEFONT, J. P., RABIER, D., CHARPENTIER, C. & KAMOUN, P. 1997. Clinical presentations of inherited mitochondrial fatty acid oxidation disorders: An update. *International Pediatrics,* 12, 34-40. | Don’t know age of diagnosis |
| SCHAEFER, J., JACKSON, S., DICK, D. J. & TURNBULL, D. M. 1996. Trifunctional enzyme deficiency: adult presentation of a usually fatal beta-oxidation defect. *Annals of Neurology,* 40, 597-602. | Not relevant |
| SCHRIJVER-WIELING, I., VAN RENS, G. H., WITTEBOL-POST, D., SMEITINK, J. A., DE JAGER, J. P., DE KLERK, H. B. & VAN LITH, G. H. 1997. Retinal dystrophy in long chain 3-hydroxy-acyl-coA dehydrogenase deficiency. *British Journal of Ophthalmology,* 81, 291-4. | Not relevant |
| SERRANO-AGUILAR, P., CASTILLA-RODRIGUEZ, I., VALLEJO-TORRES, L., VALCARCEL-NAZCO, C. & GARCIA-PEREZ, L. 2015. Neonatal screening in Spain and cost-effectiveness. *Expert Opinion on Orphan Drugs,* 3, 971-974. | Not relevant |
| SHAWKY, R. M., ABD-ELKHALEK, H. S. & ELAKHDAR, S. E. 2015. Selective screening in neonates suspected to have inborn errors of metabolism. *Egyptian Journal of Medical Human Genetics,* 16, 165-171. | Not LCHADD/TFPD |
| SHIGEMATSU, Y., HIRANO, S., HATA, I., TANAKA, Y., SUDO, M., TAJIMA, T., SAKURA, N., YAMAGUCHI, S. & TAKAYANAGI, M. 2003. Selective screening for fatty acid oxidation disorders by tandem mass spectrometry: difficulties in practical discrimination. *Journal of Chromatography B: Analytical Technologies in the Biomedical & Life Sciences,* 792, 63-72. | No LCHADD |
| SIDDIQ, S., WILSON, B. J., GRAHAM, I. D., LAMOUREUX, M., KHANGURA, S. D., TINGLEY, K., TESSIER, L., CHAKRABORTY, P., COYLE, D., DYACK, S., GILLIS, J., GREENBERG, C., HAYEEMS, R. Z., JAIN-GHAI, S., KRONICK, J. B., LABERGE, A. M., LITTLE, J., MITCHELL, J. J., PRASAD, C., SIRIWARDENA, K., SPARKES, R., SPEECHLEY, K. N., STOCKLER, S., TRAKADIS, Y., WAFA, S., WALIA, J., WILSON, K., YUSKIV, N., POTTER, B. K. & CANADIAN INHERITED METABOLIC DISEASES RESEARCH, N. 2016. Experiences of caregivers of children with inherited metabolic diseases: a qualitative study. *Orphanet Journal Of Rare Diseases,* 11, 168. | Not relevant |
| SIMS, H. F., BRACKETT, J. C., POWELL, C. K., TREEM, W. R., HALE, D. E., BENNETT, M. J., GIBSON, B., SHAPIRO, S. & STRAUSS, A. W. 1995. The molecular basis of pediatric long chain 3-hydroxyacyl-CoA dehydrogenase deficiency associated with maternal acute fatty liver of pregnancy. *Proceedings of the National Academy of Sciences of the United States of America,* 92, 841-5. | Animal study |
| SKLADAL, D., SASS, J. O., GEIGER, H., GEIGER, R., MANN, C., VREKEN, P., WANDERS, R. J. & TRAWOGER, R. 2000. Complications in early diagnosis and treatment of two infants with long-chain fatty acid beta-oxidation defects. *Journal of Pediatric Gastroenterology & Nutrition,* 31, 448-52. | 1 case |
| SOLIS, J. O. & SINGH, R. H. 2002. Management of fatty acid oxidation disorders: a survey of current treatment strategies. *Journal of the American Dietetic Association,* 102, 1800-3. | Not relevant |
| SPIEKERKOETTER, U. 2010. Mitochondrial fatty acid oxidation disorders: clinical presentation of long-chain fatty acid oxidation defects before and after newborn screening. *Journal of Inherited Metabolic Disease,* 33, 527-32. | Review |
| SPIEKERKOETTER, U., KHUCHUA, Z., YUE, Z., BENNETT, M. J. & STRAUSS, A. W. 2004. General mitochondrial trifunctional protein (TFP) deficiency as a result of either alpha- or beta-subunit mutations exhibits similar phenotypes because mutations in either subunit alter TFP complex expression and subunit turnover. *Pediatric Research,* 55, 190-6. | No outcomes |
| SPIEKERKOETTER, U., LINDNER, M., SANTER, R., GROTZKE, M., BAUMGARTNER, M. R., BOEHLES, H., DAS, A., HAASE, C., HENNERMANN, J. B., KARALL, D., DE KLERK, H., KNERR, I., KOCH, H. G., PLECKO, B., ROSCHINGER, W., SCHWAB, K. O., SCHEIBLE, D., WIJBURG, F. A., ZSCHOCKE, J., MAYATEPEK, E. & WENDEL, U. 2009. Treatment recommendations in long-chain fatty acid oxidation defects: consensus from a workshop. *Journal of Inherited Metabolic Disease,* 32, 498-505. | Review |
| SPIEKERKOETTER, U. D., SUN, B., KHUCHUA, Z., BENNETT, M. J. & STRAUSS, A. W. 2002. Molecular and phenotypic heterogeneity in mitochondrial trifunctional protein deficiency due to beta-subunit mutations. *Pediatric Research,* 51, 226A-226A. | Abstract |
| STRAUSS, A. W., SPIEKERKOETTER, U., DING, L., TOKUNAGA, C., ZYKOVITZ, T., MARSDEN, D., RINALDO, P. & BENNETT, M. 2004. The changing spectrum of fatty acid oxidation disorders post-newborn screening. *Molecular Genetics and Metabolism,* 81, 156-157. | 1 case |
| SUN, W., WANG, Y., YANG, Y., WANG, J., CAO, Y., LUO, F., LU, W., PENG, Y., YAO, H. & QIU, P. 2011. The screening of inborn errors of metabolism in sick Chinese infants by tandem mass spectrometry and gas chromatography/mass spectrometry. *Clinica Chimica Acta,* 412, 1270-4. | High risk group and no LCHADD |
| SYKUT-CEGIELSKA, J., POHORECKA, M., TAYBERT, J., GRADOWSKA, W., OLSEN, R. K. J. & ANDRESEN, B. S. 2007. Intrauterine growth retardation in patients with LCHAD deficiency. *Journal of Inherited Metabolic Disease,* 30, 50-50. | Abstract |
| TAKAHASHI, T., YAMADA, K., KOBAYASHI, H., HASEGAWA, Y., TAKETANI, T., FUKUDA, S. & YAMAGUCHI, S. 2015. Metabolic disease in 10 patients with sudden unexpected death in infancy or acute life-threatening events. *Pediatrics International,* 57, 348-53. | 1 case |
| TAKUSA, Y. & YAMAGUCHI, S. 1998. Mitochondrial trifunctional protein (TP) deficiency. [Japanese]. *Ryoikibetsu shokogun shirizu*, 422-425. | Review |
| TAL, G., PITT, J., MORRISY, S., TZANAKOS, N. & BONEH, A. 2015. An audit of newborn screening procedure: impact on infants presenting clinically before results are available. *Molecular Genetics & Metabolism,* 114, 403-8. | No LCHADD |
| TAMAOKI, Y., KIMURA, M., HASEGAWA, Y., IGA, M., INOUE, M. & YAMAGUCHI, S. 2002. A survey of Japanese patients with mitochondrial fatty acid beta-oxidation and related disorders as detected from 1985 to 2000. *Brain & Development,* 24, 675-80. | Doesn’t include age at diagnosis |
| TAUBENSLAG, L. 1972. [Congenital defects of lipid metabolism]. *Archivos Argentinos de Pediatria,* 70, 13-4. | Review |
| TEAR, F. K., HOLMSTROM, G. & YING, L. 2008. Ocular characteristics in 10 children with long-chain 3-hydroxyacyl-CoA dehydrogenase deficiency: a cross-sectional study with long-term follow-up (vol 86, pg 329, 2008). *Acta Ophthalmologica,* 86, 466-466. | Erratum |
| TERRONE, G., RUOPPOLO, M., BRUNETTI-PIERRI, N., COZZOLINO, C., SCOLAMIERO, E., PARENTI, G., ROMANO, A., ANDRIA, G., SALVATORE, F. & FRISSO, G. 2014. Child neurology: Recurrent rhabdomyolysis due to a fatty acid oxidation disorder. *Neurology,* 82, e1-4. | Single case |
| THOMASON, M. J., LORD, J., BAIN, M. D., CHALMERS, R. A., LITTLEJOHNS, P., ADDISON, G. M., WILCOX, A. H. & SEYMOUR, C. A. 1998. A systematic review of evidence for the appropriateness of neonatal screening programmes for inborn errors of metabolism. *Journal of Public Health Medicine,* 20, 331-343. | Review |
| TREEM, W. R., SHOUP, M. E., HALE, D. E., BENNETT, M. J., RINALDO, P., MILLINGTON, D. S., STANLEY, C. A., RIELY, C. A. & HYAMS, J. S. 1996. Acute fatty liver of pregnancy, hemolysis, elevated liver enzymes, and low platelets syndrome, and long chain 3-hydroxyacyl-coenzyme A dehydrogenase deficiency. *American Journal of Gastroenterology,* 91, 2293-300. | Not relevant |
| TREEM, W. R., STANLEY, C. A., HALE, D. E., LEOPOLD, H. B. & HYAMS, J. S. 1991. Hypoglycemia, hypotonia, and cardiomyopathy: The evolving clinical picture of long-chain acyl-CoA dehydrogenase deficiency. *Pediatrics,* 87, 328-333. | Single case |
| TREEM, W. R., WITZLEBEN, C. A., PICCOLI, D. A., STANLEY, C. A., HALE, D. E., COATES, P. M. & WATKINS, J. B. 1986. Medium-chain and long-chain acyl CoA dehydrogenase deficiency: clinical, pathologic and ultrastructural differentiation from Reye's syndrome. *Hepatology,* 6, 1270-8. | No age of diagnosis/method |
| TURAKA, K., BRYAN, J. S., GORDON, A. J., KWONG, H. M., JR., REDDY, R., TSIPURSKY, M. & SELL, C. H. 2012. Clinical and image-guided chorioretinal findings in long-chain 3-hydroxyacyl-coenzyme A dehydrogenase deficiency. *Journal of Pediatric Endocrinology & Metabolism,* 25, 565-7. | Abstract |
| TURNBULL, D. M., SHEPHERD, I. M. & AYNSLEY-GREEN, A. 1988. Inherited defects of mitochondrial fatty acid oxidation. *Biochemical Society transactions,* 16, 424-427. | Review |
| TYNI, T., EKHOLM, E. & PIHKO, H. 1998. Pregnancy complications are frequent in long-chain 3-hydroxyacyl- coenzyme A dehydrogenase deficiency. *American Journal of Obstetrics and Gynecology,* 178, 603-608. | No age of diagnosis/method |
| TYNI, T., IMMONEN, T., LINDAHL, P., MAJANDER, A. & KIVELA, T. 2012. Refined staging for chorioretinopathy in long-chain 3-hydroxyacyl coenzyme A dehydrogenase deficiency. *Ophthalmic Research,* 48, 75-81. | Don’t know age of diagnosis |
| TYNI, T., KIVELA, T., LAPPI, M., SUMMANEN, P., NIKOSKELAINEN, E. & PIHKO, H. 1998. Ophthalmologic findings in long-chain 3-hydroxyacyl-CoA dehydrogenase deficiency caused by the G1528C mutation - A new type of hereditary metabolic chorioretinopathy. *Ophthalmology,* 105, 810-824. | No age of diagnosis/method |
| TYNI, T., PALOTIE, A., VIINIKKA, L., VALANNE, L., SALO, M. K., VON DOBELN, U., JACKSON, S., WANDERS, R., VENIZELOS, N. & PIHKO, H. 1997. Long-chain 3-hydroxyacyl-coenzyme A dehydrogenase deficiency with the G1528C mutation: clinical presentation of thirteen patients. *Journal of Pediatrics,* 130, 67-76. | No age of diagnosis/method |
| TYNI, T. & PIHKO, H. 1997. Clinical outcomes in long-chain 3-hydroxyacyl-coenzyme A dehydrogenase deficiency - Reply. *Journal of Pediatrics,* 131, 938-+. | Reply |
| TYNI, T., RAPOLA, J., PAETAU, A., PALOTIE, A. & PIHKO, H. 1997. Pathology of long-chain 3-hydroxyacyl-CoA dehydrogenase deficiency caused by the G1528C mutation. *Pediatric Pathology & Laboratory Medicine,* 17, 427-47. | All screened late |
| USHIKUBO, S., AOYAMA, T., KAMIJO, T., WANDERS, R. J., RINALDO, P., VOCKLEY, J. & HASHIMOTO, T. 1996. Molecular characterization of mitochondrial trifunctional protein deficiency: formation of the enzyme complex is important for stabilization of both alpha- and beta-subunits. *American Journal of Human Genetics,* 58, 979-88. | No age of diagnosis/method |
| UUSIMAA, J., VAINIONPAA, L., SIMILA, S., MIETTINEN, R. & NUUTINEN, M. 1997. L-3-Hydroxyacyl-CoA dehydrogenase deficiency: Two cases with pigmentary retinopathy. *Journal of Inherited Metabolic Disease,* 20, 848-850. | 2 cases both late |
| VALAYANNOPOULOS, V., BARNERIAS, C., ROMANO, S., BRIVET, M., VIANNEY-SABAN, C., DESGUERRE, I., TOUATI, G., SAUDUBRAY, J. M. & DE LONLAY, P. 2006. Peripheral neuropathy as a presenting symptom in mitochondrial trifunctional protein (MTP) deficiency. *Journal of Inherited Metabolic Disease,* 29, 56-56. | Abstract |
| VAN GRUNSVEN, E. G., VAN ROERMUND, C. W. T., DENIS, S. & WANDERS, R. J. A. 1997. Complementation analysis of fibroblasts from peroxisomal fatty acid oxidation deficient patients shows high frequency of bifunctional enzyme deficiency plus intragenic complementation: Unequivocal evidence for differential defects in the same enzyme protein. *Biochemical and Biophysical Research Communications,* 235, 176-179. | No outcomes |
| VAN HOVE, J. L. K., KAHLER, S. G., FEEZOR, M. D., RAMAKRISHNA, J. P., HART, P., TREEM, W. R., SHEN, J. J., MATERN, D. & MILLINGTON, D. S. 2000. Acylcarnitines in plasma and blood spots of patients with long-chain 3-hydroxyacyl-coenzyme A dehydrogenase deficiency. *Journal of Inherited Metabolic Disease,* 23, 571-582. | No outcomes |
| VAN MALDERGEM, L., TUERLINCKX, D., WANDERS, R. J., VIANEY-SABAN, C., VAN HOOF, F., MARTIN, J. J., FOURNEAU, C., GILLEROT, Y. & BACHY, A. 2000. Long-chain 3-hydroxyacyl-CoA dehydrogenase deficiency and early-onset liver cirrhosis in two siblings. *European Journal of Pediatrics,* 159, 108-12. | No age of diagnosis/method |
| VENTURA, F. V., RUITER, J. P. N., IJLST, L., DE ALMEIDA, I. T. & WANDERS, R. J. A. 1998. Lactic acidosis in long-chain fatty acid beta-oxidation disorders. *Journal of Inherited Metabolic Disease,* 21, 645-654. | Cell study |
| VIANEY-LIAUD, C., DIVRY, P., GREGERSEN, N. & MATHIEU, M. 1987. The inborn errors of mitochondrial fatty acid oxidation. *Journal of Inherited Metabolic Disease,* 10 Suppl 1, 159-200. | Review |
| VICI, C. D., BERTINI, E., BURLINA, A., GARAVAGLIA, B., HALE, D. E., BARTULI, A., MAZZIOTTA, M. R. M., SABATELLI, M. & SABETTA, G. 1990. NEUROMUSCULAR INVOLVEMENT IN 2 UNRELATED CHILDREN WITH LONG-CHAIN 3-HYDROXYACYL-COA DEHYDROGENASE (LCHAD) DEFICIENCY. *Pediatric Research,* 28, 305-305. | Abstract |
| VILASECA, M. A., GOMEZ-LOPEZ, L., LAMBRUSCHINI, N., GUTIERREZ, A., GARCIA, R., MEAVILLA, S., MORENO, J. & ARTUCH, R. 2011. Long-chain polyunsaturated fatty acid concentration in patients with inborn errors of metabolism. *Nutricion Hospitalaria,* 26, 128-136. | No LCHADD |
| VIST, G. E., FRONSDAL, K. B., JOHANSEN, M., HOFMANN, B. & FRETHEIM, A. 2007. *Knowledge Centre for the Health Services at The Norwegian Institute of Public Health (NIPH),* NIPH Systematic Reviews, Executive Summaries. | Review |
| VOCKLEY, J., BURTON, B., BERRY, G. T., LONGO, N., PHILLIPS, J., SANCHEZ-VALLE, A., TANPAIBOON, P., GRUNEWALD, S., MURPHY, E., BOWDEN, A., CHEN, W. C., MU, Y. M., CATALDO, J., MARSDEN, D. & KAKKIS, E. 2018. RESULTS FROM A 78-WEEK SINGLE-ARM, OPEN-LABEL PHASE 2 STUDY TO EVALUATE UX007 IN PEDIATRIC AND ADULT PATIENTS WITH MODERATE TO SEVERE LONG-CHAIN FATTY ACID OXIDATION DISORDERS (LC-FAOD). *Molecular Genetics and Metabolism,* 123, 274-275. | Abstract |
| VOCKLEY, J., BURTON, B., BERRY, G. T., LONGO, N., PHILLIPS, J., SANCHEZ-VALLE, A., TANPAIBOON, P., GRUNEWALD, S., MURPHY, E., HUMPHREY, R., MAYHEW, J., BOWDEN, A., ZHANG, L., CATALDO, J., MARSDEN, D. L. & KAKKIS, E. 2017. UX007 for the treatment of long chain-fatty acid oxidation disorders: Safety and efficacy in children and adults following 24weeks of treatment. *Molecular Genetics & Metabolism,* 120, 370-377. | No age at diagnosis, can’t separate from other conditions |
| VOCKLEY, J., MARSDEN, D., MCCRACKEN, E., DEWARD, S., BARONE, A., HSU, K. & KAKKIS, E. 2015. Long-term major clinical outcomes in patients with long chain fatty acid oxidation disorders before and after transition to triheptanoin treatment--A retrospective chart review. *Molecular Genetics & Metabolism,* 116, 53-60. | Don’t know age at diagnosis |
| VREKEN, P., VAN LINT, A. E., BOOTSMA, A. H., OVERMARS, H., WANDERS, R. J. & VAN GENNIP, A. H. 1999. Quantitative plasma acylcarnitine analysis using electrospray tandem mass spectrometry for the diagnosis of organic acidaemias and fatty acid oxidation defects. *Journal of Inherited Metabolic Disease,* 22, 302-6. | Not relevant |
| WAISBREN, S. E., HE, J. & MCCARTER, R. 2015. Assessing Psychological Functioning in Metabolic Disorders: Validation of the Adaptive Behavior Assessment System, Second Edition (ABAS-II), and the Behavior Rating Inventory of Executive Function (BRIEF) for Identification of Individuals at Risk. *Jimd Reports,* 21, 35-43. | Don’t know age or method of diagnosis |
| WAJNER, M., COELHO DDE, M., INGRASSIA, R., DE OLIVEIRA, A. B., BUSANELLO, E. N., RAYMOND, K., FLORES PIRES, R., DE SOUZA, C. F., GIUGLIANI, R. & VARGAS, C. R. 2009. Selective screening for organic acidemias by urine organic acid GC-MS analysis in Brazil: fifteen-year experience. *Clinica Chimica Acta,* 400, 77-81. | Not relevant |
| WANDERS, R. J., IJLST, L., DURAN, M., JAKOBS, C., DE KLERK, J. B., PRZYREMBEL, H., ROCCHICCIOLI, F. & AUBOURG, P. 1991. Long-chain 3-hydroxyacyl-CoA dehydrogenase deficiency: different clinical expression in three unrelated patients. *Journal of Inherited Metabolic Disease,* 14, 325-8. | Both late cases |
| WANDERS, R. J., VREKEN, P., DEN BOER, M. E., WIJBURG, F. A., VAN GENNIP, A. H. & L, I. J. 1999. Disorders of mitochondrial fatty acyl-CoA beta-oxidation. *Journal of Inherited Metabolic Disease,* 22, 442-87. | Review |
| WANDERS, R. J. A., IJLST, L., VANGENNIP, A. H., JAKOBS, C., DEJAGER, J. P., DORLAND, L., VANSPRANG, F. J. & DURAN, M. 1990. LONG-CHAIN 3-HYDROXYACYL-COA DEHYDROGENASE-DEFICIENCY - IDENTIFICATION OF A NEW INBORN ERROR OF MITOCHONDRIAL FATTY-ACID BETA-OXIDATION. *Journal of Inherited Metabolic Disease,* 13, 311-314. | Case study |
| WASANT, P., SVASTI, J., SRISOMSAP, C., LIAMMONGKOLKUL, S., NAYLOR, E. W. & MATSUMOTO, I. 1999. Inherited metabolic disorders in Thailand--Siriraj experience. *Southeast Asian Journal of Tropical Medicine & Public Health,* 30 Suppl 2, 124-37. | No LCHADD – case study |
| WILCKEN, B. 2010. Expanded newborn screening: reducing harm, assessing benefit. *Journal of Inherited Metabolic Disease,* 33, S205-10. | Review |
| WILCKEN, B. 2010. Fatty acid oxidation disorders: outcome and long-term prognosis. *Journal of Inherited Metabolic Disease,* 33, 501-6. | Review |
| WILCKEN, B., LEUNG, K. C., HAMMOND, J., KAMATH, R. & LEONARD, J. V. 1993. Pregnancy and fetal long-chain 3-hydroxyacyl coenzyme A dehydrogenase deficiency. *Lancet,* 341, 407-8. | All early |
| WILCOX, R. L., NELSON, C. C., STENZEL, P. & STEINER, R. D. 2002. Postmortem screening for fatty acid oxidation disorders by analysis of Guthrie cards with tandem mass spectrometry in sudden unexpected death in infancy. *Journal of Pediatrics,* 141, 833-6. | No LCHADD |
| WILEY, V., CARPENTER, K., BAYLISS, U. & WILCKEN, B. 2003. Newborn screening--is it really that simple? *Southeast Asian Journal of Tropical Medicine & Public Health,* 34 Suppl 3, 107-10. | No results for LCHADD |
| WILSON, C., KNOLL, D., DE HORA, M., KYLE, C., GLAMUZINA, E. & WEBSTER, D. 2017. The Risk of Fatty Acid Oxidation Disorders and Organic Acidemias in Children with Normal Newborn Screening. *Jimd Reports,* 35, 53-58. | Not LCHADD |
| XIE, L. J., ZHU, J. X., ZHU, X. D., LI, H. J., HAN, L. S. & GU, X. F. 2008. Combined use of tandem mass spectrometry with urine gas chromatography/mass spectrometry is useful for diagnosis of inborn errors of metabolism in children. [Chinese]. *Chinese Journal of Contemporary Pediatrics,* 10, 31-34. | Not relevant |
| YAMADA, K., HASEGAWA, Y., YOSHIKAWA, Y., TAKAHASHI, T., KOBAYASHI, H., MUSHIMOTO, Y., PUREVSUREN, J. & YAMAGUCHI, S. 2013. Clinical study of organic acidemias and fatty acid oxidation disorders detected in adults. [Japanese]. *Clinical Neurology,* 53, 191-195. | Not LCHADD |
| YAMAGUCHI, S. 2001. Acyl-CoA dehydrogenase deficiency (very-long-chain, long-chain, medium-chain, short-chain), mitochondrial. [Japanese]. *Ryoikibetsu shokogun shirizu*, 144-147. | Review |
| YAMAGUCHI, S. 2002. [Enzymes of mitochondrial beta-oxidation]. *Nippon Rinsho - Japanese Journal of Clinical Medicine,* 60 Suppl 4, 88-93. | Review |
| YAMAGUCHI, S. 2002. Mitochondrial 3-hydroxyacyl-CoA dehydrogenase (SCHAD, LCHAD). [Japanese]. *Nippon rinsho,* Japanese journal of clinical medicine. 60 Suppl 4, 101-104. | Review |
| YAMAMOTO, T., MISHIMA, H., MIZUKAMI, H., FUKAHORI, Y., UMEHARA, T., MURASE, T., KOBAYASHI, M., MORI, S., NAGAI, T., FUKUNAGA, T., YAMAGUCHI, S., YOSHIURA, K.-I. & IKEMATSU, K. 2015. Metabolic autopsy with next generation sequencing in sudden unexpected death in infancy: Postmortem diagnosis of fatty acid oxidation disorders. *Molecular Genetics and Metabolism Reports,* 5, 26-32. | Not relevant |
| YANG, Z., LANTZ, P. E. & IBDAH, J. A. 2007. Post-mortem analysis for two prevalent beta-oxidation mutations in sudden infant death. *Pediatrics International,* 49, 883-7. | 1 case |
| ZHENG, J., ZHANG, Y., HONG, F., YANG, J., TONG, F., MAO, H., HUANG, X., ZHOU, X., YANG, R., ZHAO, Z. & HUANG, X. 2017. [Screening for fatty acid oxidation disorders of newborns in Zhejiang province:prevalence, outcome and follow-up]. *Zhejiang da Xue Xue Bao Yi Xue Ban/Journal of Zhejiang University Medical Sciences,* 46, 248-255. | No LCHADD |
| ZHU, J. & YANG, Z. 2006. Study of the inborn errors of mitochondrial fatty acid beta-oxidation deficiency. [Chinese]. *Beijing da xue xue bao,* Yi xue ban = Journal of Peking University. Health sciences. 38, 214-217. | Review |
| ZHU, J. M., YANG, Z., YU, M., WANG, R., YE, R. H., YANG, H. X., ZHAI, G. R. & WANG, Q. 2005. Screening for the G1528C mutation in long chain fatty acid oxidation enzyme in Han nationality in Beijing population. [Chinese]. *Beijing da xue xue bao,* Yi xue ban = Journal of Peking University. Health sciences. 37, 72-74. | No LCHADD |
