# Supplementary material for: Evaluation of earlier versus later dietary management in long-chain 3-hydroxyacyl-CoA dehydrogenase or mitochondrial trifunctional protein deficiency: a systematic review
Source: Orphanet J Rare Dis. 2019 Nov 15;14:258. doi: 10.1186/s13023-019-1226-y (PMC6858661; doi:10.1186/s13023-019-1226-y)
Supplement: Supplementary file 4 — Additional file 4. Methodological quality of included studies. [file 13023_2019_1226_MOESM4_ESM.docx]

Additional file 4: Study quality of included studies according to EPHPP quality assessment tool

| **Study** | **Global rating from sections A-F** | | | | | | **Global rating for this study** |
| --- | --- | --- | --- | --- | --- | --- | --- |
|  | **A)**  **Selection bias** | **B)**  **Study design** | **C)**  **Confounders** | **D)**  **Blinding** | **E)**  **Data collection methods** | **F)**  **Withdrawals and drop-outs** |  |
| **Swedish study** |  |  |  |  |  |  |  |
| Fahnehjelm 2008 [49] | Moderate | Moderate | Weak | Moderate | Moderate | Strong | Weak |
| Haglind 2013 [51] | Moderate | Moderate | Weak | Moderate | Moderate | Strong | Moderate |
| Fahnehjelm 2016 [50] | Moderate | Moderate | Weak | Moderate | Moderate | Strong | Weak |
| **European single centre studies** |  |  |  |  |  |  |  |
| Immonen 2015 [31] | Strong | Moderate | Weak | Moderate | Weak | Strong | Weak |
| Sperk 2010 [38] | Strong | Moderate | Weak | Moderate | Weak | Strong | Weak |
| Gillingham 2017 [28] | Weak | Moderate | Weak | Moderate | weak | Strong | Weak |
| **European collaboration studies** |  |  |  |  |  |  |  |
| Karall 2015 [52] | Moderate | Moderate | Weak | Moderate | Moderate | Strong | Moderate |
| Spiekerkoetter 2009 [41] | weak | Moderate | Weak | Moderate | Weak | moderate | Weak |
| **Non-European single centre studies** |  |  |  |  |  |  |  |
| Boese 2016 [23] | Strong | Moderate | Weak | Moderate | Moderate | Strong | Moderate |
| De Biase 2017 [26] | Weak | Moderate | Weak | Moderate | Weak | Strong | Weak |
| Kang 2018 [33] | strong | Moderate | Weak | Moderate | Moderate | Strong | Moderate |
| **European collaboration studies** |  |  |  |  |  |  |  |
| Lund 2012 [6] | Strong | Moderate | weak | Moderate | Moderate | Strong | Moderate |
| Sykut-Cegielska 2011 [7] | Strong | Moderate | Weak | Moderate | Weak | Strong | Weak |
