# Supplementary material for: Evaluation of earlier versus later dietary management in long-chain 3-hydroxyacyl-CoA dehydrogenase or mitochondrial trifunctional protein deficiency: a systematic review
Source: Orphanet J Rare Dis. 2019 Nov 15;14:258. doi: 10.1186/s13023-019-1226-y (PMC6858661; doi:10.1186/s13023-019-1226-y)
Supplement: Supplementary file 5 — Additional file 5. Follow up analyses. [file 13023_2019_1226_MOESM5_ESM.docx]

**Additional file 5 – Follow up analyses**

| **Follow up analyses** | **Mortality** | **Heart problems** | **Liver problems** | **Visual problems** | **Neurological problems** | **Motor/muscular** | **Hypoglycaemia** |
| --- | --- | --- | --- | --- | --- | --- | --- |
| 1) Asympto-matic vs symptomatic | Immonen 2016  0/1 vs 6/15 (p=1)  Sperk 2010  1/3 vs 0/3 (p=1)  Kang 2018  1/1 vs 2/6 (p=1)  Lund 2012  0/3 vs 1/2  (p=0.4)  Skyut-Cegielska 2011  1/15 vs 19/44  (p<0.05)  Spiekerkoetter 2009  0/7 vs 8/20  (p=0.17) | **Cardiomyo-pathy**  Immonen 2016  Start 0/1 vs 6/10  (p=0.46)  End 0/1 vs 4/10  (p=0.64)  Sperk 2010  0/3 vs 2/3 (p=0.4)  Karall 2015  1/6 vs 6/8 (p=1)  Lund 2012  0/3 vs 2/2 (p=1)  **Arrhythmias**  De Biase 2017  0/1 vs 1/4 (p=1)  **Cardiac complications**  Gillingham 2017  0/7 vs 3/5  (p<0.05) | **Hepatopathy**  Karall 2015  1/6 vs 4/8  (p=0.4)  Lund 2012  0/3 vs 1/2  (p=0.4) | **ERG findings**  Swedish cohort  Fahnehjelm 2016  Normal vs subnormal/pathological  1/2 vs 8/9 (p=0.35)  **Ocular Fundii**  Normal vs subnormal/pathological/ severely pathological  2/3 vs 9/9 (p=0.25)  **Photophobia**  Swedish cohort  Fahnehjelm 2008  1/1 vs 7/8 (p=1)  **Nyctalopia**  Swedish cohort  Fahnehjelm 2008  0/1 vs 2/8 (p=1)  **Retinopathy**  Immonen 2016  0/1 vs 9/10 (p=0.22)  Karall 2015  2/6 vs 6/8 (p=0.28)  De Biase 2017  1/1 vs 4/5 (p=1) | **Epilepsia**  Swedish Cohort  Fahnehjelm 2008  0/3 vs 3/8  (p=0.51)  **Neurological symptoms**  De Biase 2017  0/1 vs 1/4  (p=1) | **Psychomotor development**  Swedish Cohort  Fahnehjelm 2008[2]  0/1 vs 5/9  (p=1)  Karall 2015  0/6 vs 0/8  NA  **Myopathy**  Sperk 2010  0/3 vs 1/3  (p=1)  De Biase 2017  0/1 vs 1/4  (p=1)  **>1 episode of rhabdomyolysis**  Boese 2016  5/6 vs 14/14  (p=0.3) | Swedish cohort study Fahnehjelm 2016  1/3 vs 7/9 (p=0.27)  Sperk 2010  1/3 vs 3/3 (p=0.4) |
| 2) Screened vs unscreened | Lund 2012  0/3 vs 1/2  (p=0.4)  Immonen 2016  0/1 vs 6/15  (p=1)  Kang 2018  1/1 vs 2/6 (p=1)  Spiekerkoetter 2009  2/10 vs 6/17  (p=0.67)  Skyut-Cegielska 2011  1/15 vs 13/37  (p<0.05) | **Cardiomyo-pathy**  Karall 2015  2/9 vs 5/5 (p=0.02)  Lund 2012  0/3 vs 2/2 (p=1)  Immonen 2016  Start 0/1 vs 6/10  (p=0.46)  End 0/1 vs 4/10  (p=0.64)  Spiekerkoetter 2009  4/10 vs 8/17 (p=1)  **Arrhythmias**  De Biase 2017  0/3 vs 1/2 (p=0.4)  **Cardiac complications**  Gillingham 2017  0/7 vs 3/5  (p<0.05) | **Hepatopathy**  Karall 2015  1/9 vs 4/5  (p=0.02)  Lund 2012  0/3 vs 1/2  (p=0.4)  **Reye syndrome**  Spiekerkoetter 2009  3/10 vs 6/17  (p=0.69) | **ERG findings**  Swedish cohort  Fahnehjelm 2016  Normal vs subnormal/pathological  1/2 vs 8/9 (p=0.35)  **Ocular Fundii**  Normal vs subnormal/pathological/ severely pathological  2/3 vs 9/9 (p=0.25)  **Photophobia**  Swedish cohort  Fahnehjelm 2008  1/1 vs 7/8 (p=1)  **Nyctalopia**  Swedish cohort  Fahnehjelm 2008  0/1 vs 2/8 (p=1)  **Retinopathy**  Karall 2015  3/9 vs 5/5 (p=0.03)  De Biase 2017  2/3 vs 2/2 (p=1)  Immonen 2016 0/1 vs 9/10 (p=0.22)  **Vision^a^**  Boese 2016  Normal visit 1:  7/7 vs 14/14 (p=NA)  Normal Visit 2:  7/7 vs 12/14  (p=0.53) | **Epilepsia**  Swedish Cohort  Fahnehjelm 2008  0/3 vs 3/8  (p=0.51)  **Neurological symptoms**  De Biase 2017  0/3 vs 1/2  (p=0.4) | **Psychomotor development**  Swedish Cohort  Fahnehjelm 2008  0/1 vs 5/9  (p=1)  **Myoglobinuria**  De Biase 2017  0/3 vs 1/2  (p=0.4)  **Myopathy/Hypotonia**  Spiekerkoetter 2009  4/10 vs 14/17  (p=0.03)  **>1 episode of rhabdomyolysis**  Boese 2016  5/6 vs 14/14 (p=0.3) | Swedish cohort study Fahnehjelm 2016  1/3 vs 7/9 (p=0.27)  Spiekerkoetter 2009  4/10 vs 15/17  (p=0.02) |
| 3) Asympto-matic screened, symptomatic screened or symptomatic clinically diagnosed | Spiekerkoetter 2009  0/4 vs 2/6 vs 6/17 (p=0.42) | **Cardiomyo-pathy**  Karall 2015  1/6 vs 1/3 vs 5/5  (p<0.05)  **Arrhythmias**  De Biase 2017  0/1 vs 0/2 vs 1/2  (p=1) | **Hepatopathy**  Karall 2015  1/6 vs 0/3 vs 4/5  (p=0.06) | **Retinopathy**  Karall 2015  1/6 vs 1/3 vs 4/5 (p=0.05)  De Biase 2017  1/1 vs 1/2 vs 2/2 (p=1)  **Best corrected visual acuity**  Boese 2016  Central steady and maintained  Visit 1:  3/6 vs 1/1 vs 4/14 (p=0.33)  Visit 2:  0/6 vs 0/1 vs 2/14 (p=1)  **Vision^a^**  Boese 2016  Normal visit 1:  6/6 vs 1/1 vs 14/14  (P value NA)  Normal Visit 2:  6/6 vs 1/1 vs 12/14 (p=1) | **Neurological symptoms**  De Biase 2017  0/1 vs 0/2 vs 1/2  (p=1) | **Myoglobinuria**  De Biase 2017  0/1 vs 0/2 vs 1/2  (p=1) | **Hypoglycaemia at diagnosis**  De Biase 2017  0/1 vs 2/2 vs 1/2  (p=0.6) |

VS versus; NA not applicable ERG, electroretinography; LCHADD, long-chain 3-hydroxyacyl-CoA dehydrogenase deficiency; MTPD, Mitochondrial Trifunctional Protein Disorder

a Vision calculated by reviewers. The World Health Organization established criteria for low vision using the LogMAR scale. Low vision is defined as a best-corrected visual acuity worse than 0.5 LogMAR but equal or better than 1.3 LogMAR in the better eye. Blindness is defined as a best-corrected visual acuity worse than 1.3 LogMAR in the better eye. Normal defined as above 0.5
